# A Novel Role of Neutrophil Elastase in Podocyte Dysfunction Induced by High Glucose, PMA, and MDP

**DOI:** 10.1002/jcp.70116

**Published:** 2025-12-03

**Authors:** Tomasz Kulesza, Aleksandra Wróblewska, Justyna Sawicka, Irena Audzeyenka, Patrycja Rachubik, Dorota Rogacka, Wiktoria Mallek, Magdalena Wysocka, Adam Lesner, Agnieszka Piwkowska

**Affiliations:** ^1^ Laboratory of Molecular Enzymology and Oncology, Intercollegiate Faculty of Biotechnology Medical University of Gdansk and University of Gdansk Gdansk Pomorskie Poland; ^2^ Laboratory of Molecular and Cellular Nephrology Mossakowski Medical Research Institute, Polish Academy of Sciences Gdansk Pomorskie Poland; ^3^ Doctoral School of Translational Medicine Centre of Postgraduate Medical Education Warsaw Mazowieckie Poland; ^4^ Department of Biomedical Chemistry, Faculty of Chemistry University of Gdansk Gdansk Pomorskie Poland; ^5^ Department of Environmental Technology, Faculty of Chemistry University of Gdansk Gdansk Pomorskie Poland

**Keywords:** diabetic kidney disease, insulin resistance, neutrophil elastase, neutrophil serine proteases, NOD2, podocyte

## Abstract

Podocytes are not only the key regulators of glomerular filtration barrier dynamics but also exhibit immunological properties. They are capable of antigen presentation and possess a receptor system recognizing pathogen‐associated molecular patterns. Our earlier study further demonstrated that podocytes share additional similarities with immune cells, as they can synthesize and secrete the active form of cathepsin C – an enzyme that controls the activation of neutrophil serine proteases (NSPs). In this study, we established that podocytes synthesize neutrophil elastase (NE), proteinase 3 (PR 3), and cathepsin G (CatG) but also release their active forms into the extracellular environment. We found that NSPs contribute to podocyte dysfunction upon inflammation induction by PMA and under conditions of insulin insensitivity. Moreover, actin cytoskeleton rearrangement and increased albumin permeability of the podocyte monolayer were triggered by nucleotide‐binding and oligomerization domain‐containing protein 2 (NOD2) activation via muramyl dipeptide (MDP), which consequently enhanced NE and PR 3 activity. Notably, silencing the *ELANE* gene, which encodes NE, exerted a protective effect on podocytes even after NOD2 activation. These findings indicate that NSPs, especially NE, contribute to podocyte dysfunction in diabetes and diabetic kidney disease, a condition characterized by chronic inflammation and insulin resistance.

## Graphical abstract

Podocyte injury occurs as a result of neutrophil elastase activity, a process in which NOD2 plays a contributing role.

**Abbreviations**
CatG: cathepsin G
FBS: fetal bovine serum
GFB: glomerular filtration barrier
GLUT4: glucose transporter type 4
HG: high glucose medium
HSP90: heat shock protein 90
IKK: inhibitor of NF‐κB kinase
MDP: muramyl dipeptide
MNEI: monocyte neutrophil elastase inhibitor
NE: neutrophil elastase
NETs: neutrophil extracellular traps
NF‐κB: nuclear factor‐kappa B
NG: normal glucose medium
NLR: NOD‐like receptor
NOD2: nucleotide‐binding and oligomerization domain containing protein 2
NSPs: neutrophil serine proteases
PMA: phorbol 12 myristate‐13‐acetate
PR 3: proteinase 3
ROS: reactive oxygen species
SOCS3: suppressor of cytokine signaling 3
TLR: toll‐like receptor

## 1 Introduction

The renal glomerulus is a remarkable system with exceptional efficiency, as it enables the formation of approximately 180 liters of primary urine daily (Kaufman et al. 2023). However, due to the continuous blood flow through the capillaries, the delicate structures of the glomerulus are constantly exposed to pathogenic and autoimmune factors. Glomerular ultrafiltration occurs due to a highly selective molecular sieve, known as the glomerular filtration barrier (GFB), which consists of fenestrated endothelium, the glomerular basement membrane, and the foot processes of podocytes that envelop it, forming the slit diaphragm (Pollak et al. 2014). The aforementioned podocytes are post‐mitotic epithelial cells, and their irreversible loss frequently contributes to the development of glomerular sclerosis and overt proteinuria (Faul 2014). As insulin‐dependent cells, podocytes experience functional impairment in the course of diabetes or metabolic syndrome (Han et al. 2013; Jauregui et al. 2009).

In addition to the increasingly well‐characterized effects of hyperglycemia on podocyte function, in recent years, numerous researchers have emphasized the role of protease activity in impairing the functionality of the GFB (Garsen et al. 2016; Guan et al. 2017; Höhne et al. 2018; Sever et al. 2007). It is worth mentioning that podocytes themselves possess a complex network of proteolytic enzymes (Rinschen et al. 2018). The involvement of cathepsins in the development of glomerulopathy deserves particular attention. The activity of the cysteine protease cathepsin L leads to the degradation of podocyte‐associated proteins closely linked to actin cytoskeleton dynamics (CD2AP, synaptopodin, dynamin), ultimately resulting in podocyte loss through urine (Sever et al. 2007; Yaddanapudi et al. 2011). In contrast, cathepsin D exhibits a protective effect on podocytes, as its downregulation leads to GFB disruption and proteinuria (Yamamoto‐Nonaka et al. 2016). Furthermore, in our earlier study, we established that podocytes are able to produce and secrete cathepsin C, whose activity increases in a hyperglycemic (HG) environment (Audzeyenka et al. 2020). This phenomenon led to cytoskeletal rearrangement and elevated albumin permeability. It is worth noting that cathepsin C plays a superior role over neutrophil serine proteases (NSPs), which are synthesized as inactive zymogens and stored in neutrophil granules. The catalytic activity of cathepsin C enables the activation of NSPs, which include neutrophil elastase (NE), proteinase 3 (PR 3), and cathepsin G (CatG) (Chalmers et al. 2023). NSPs are key components of neutrophil extracellular traps (NETs), which are released by activated neutrophils and serve as both a decoy and a degradation site for extracellular pathogens. In addition to their protective role, increasing evidence suggests that NETs contribute to the development of various systemic diseases (Mutua and Gershwin 2021; Y. Wang et al. 2014). It is also important to highlight that NSPs play a considerable role in the dysregulation of glucose homeostasis and its consequences. Both NE and PR 3 activity is elevated in the serum of patients with type 2 diabetes (Mirea et al. 2019). Additionally, NE is believed to participate in the development of diabetic retinopathy (Lessieur et al. 2021). These activated enzymes are secreted during neutrophil degranulation, which is accompanied by reactive oxygen species (ROS) generation via NADPH oxidase activity. The above‐mentioned mechanisms primarily contribute to glomerular damage associated with neutrophil infiltration (Feith et al. 1996; J. Z. Li et al. 1994). During neutrophil degranulation, not only NSPs but also their endogenous inhibitors are released into the extracellular environment. One such inhibitor is monocyte neutrophil elastase inhibitor (MNEI, also known as Serpin B1), which is among the most potent and specific protease inhibitors that irreversibly bind to the active site of NE (Bronze‐Da‐Rocha and Santos‐Silva 2018).

Recruitment of ROS‐producing immune cells (neutrophils and macrophages) to the glomerulus is a prolonged process lasting from several hours up to a full day (Gluhovschi et al. 2010). Podocytes are continuously exposed to pathogenic factors present in the blood flowing through the glomerulus. Consequently, they have developed defense mechanisms characteristic of immune cells, allowing them to respond before leukocyte infiltration occurs at the site of injury. Among these adaptations is the presence of MHC class I and II molecules, which enable the phagocytosis of pathogens and the presentation of their components on the cell surface (Goldwich et al. 2013; S. Li et al. 2020). Secondly, podocytes express pattern recognition receptors, including toll‐like receptors (TLR), NOD‐like receptors (NLR), and NLRP inflammasome, which mediate between identification of the threat and exocytosis of pro‐inflammatory cytokines (L. Y. Wang et al. 2019). Of particular note should be the nucleotide‐binding and oligomerization domain containing protein 2 (NOD2), which is activated by muramyl dipeptide (MDP), a component of the cell wall of Gram‐positive and Gram‐negative bacteria (Keestra‐Gounder and Tsolis 2017). It has been established that direct activation of NOD2 elevates the activity of NSPs, including NE (Alyami et al. 2019). Beyond detecting MDP and triggering the pro‐inflammatory response, NOD2 protein has recently been associated with the development of Crohn′s disease, as well as oxidative stress and insulin resistance (Denou et al. 2015; Maurya et al. 2015; Mohanan and Grimes 2014; Tamrakar et al. 2010). There are also reports on the association between vimentin—an intermediate filament component of the cytoskeleton—and NOD2's ability to activate the nuclear factor‐kappa B (NF‐κB)‐dependent pathway (Stevens et al. 2013). Notably, vimentin upregulation serves as an early marker of podocyte injury (Funk et al. 2016).

Delving further into the research, we arrived at the conclusion that the involvement of NOD2 in the progression of kidney damage, specifically targeting podocytes, ought to be mentioned. Three studies are available that directly examined the function of NOD2 in podocytes. Firstly, NOD2 activity induced the overexpression of the TRPC6 calcium channel, leading to exaggerated intracellular calcium influx, podocyte cytoskeletal redistribution, and their apoptosis (Han et al. 2013). The next two studies focus on the relationship between the diabetic environment and NOD2 distribution: (a) NOD2 initiated an inflammatory response while simultaneously impairing insulin signaling and reducing insulin‐driven glucose uptake in podocytes (Du et al. 2013); (b) NOD2 stimulation triggers NF‐κB‐mediated signaling, resulting in the release of potent pro‐inflammatory cytokines such as IL‐1β, IL‐6, IL‐18, and TNF‐α by podocytes (Y. Wang et al. 2024).

Given these relationships, our group aimed to investigate how inflammation induced by PMA or MDP influences the expression and activity of NSPs in human podocytes that have lost insulin sensitivity. To further examine the involvement of NE in podocyte injury, we established a podocyte cell line with lentivirus‐mediated silencing of the *ELANE* gene, which encodes NE, enabling a more in‐depth analysis of its functional significance.

## 2 Materials and Methods

### 2.1 Podocyte Cell Line

Immortalized human podocyte cell line (RRID: CVCL_W186) was a kind gift of Prof. Moin A. Saleem (Saleem et al. 2002). Purity of the cell line was checked regularly as described previously (Audzeyenka et al. 2020). Podocytes were cultured at 33°C, 5% CO_2_ in 11 mM glucose RPMI 1640 medium (Catalog No. 21875059; Thermo Fisher Scientific) supplemented with 10% fetal bovine serum (FBS; Catalog No. 10270106; Thermo Fisher Scientific), 100 U/mL penicillin, and 100 mg/mL streptomycin (Catalog No. 15140122; Thermo Fisher Scientific). Then, after reaching the desired confluence, the cells were incubated at 37°C, 5% CO_2_ in RPMI 1640 (Catalog No. 01‐101‐1; Sartorius) supplemented with 5.6 mM glucose, FBS, and antibiotics for 10–14 days until total differentiation. Next, the cells were cultured in normal glucose (5.6 mM) medium, which served as a control or high glucose (30 mM, HG) medium for 5 days, known to cause insensitivity of podocytes to insulin (Rogacka et al. 2014). To induce inflammatory processes, 100 nM phorbol‐12‐myristate‐13‐acetate (PMA; Catalog No. sc‐3576A; Santa Cruz Biotechnology) was added for 24 h to the culture medium. PMA serves as a potent activator of protein kinase C (Ambrus et al. 2015). To activate NOD2, 10 µg/ml muramyl dipeptide (MDP; Catalog No. tlrl‐mdp; Invivogen) was also used for 24 h.

### 2.2 Lentiviral Transduction and Gene Silencing

The human podocyte cell line with silenced *ELANE* gene, which encodes neutrophil elastase, was generated. Cells were transduced with GIPZ *ELANE* short hairpin RNA (shRNA) viral particles (shELANE) and GIPZ nonsilencing shRNA viral particles (Control; Dharmacon), which served as a negative control. To intensify the transduction efficiency, polybrene (Catalog No. H9268; Sigma‐Aldrich) was added to dividing podocytes. Puromycin was used to perform the selection of shRNA‐expressing podocytes.

### 2.3 Western Blot

Cell lysates were generated using RIPA Lysis Buffer (Catalog No. 20‐188; Merck). Western blot procedure was performed as described earlier (Kulesza et al. 2022). The primary antibodies used are listed in Table 1. Densitometry evaluation of the resulting bands was performed using Image J software (National Institutes of Health).

**Table 1:** Primary antibodies used in the experiments.

Antibody	Dilution	Source
β‐actin	1:10,000	Sigma‐Aldrich; Catalog No. A5441 (WB)
Serpin B1	1:800	Santa Cruz Biotechnology; Catalog No. sc‐293462 (WB)
NE	1:10,000	Abclonal; Catalog No. A13015 (WB)
NOD2	1:50	Merck; Catalog No. 04‐145 (IF)

Abbreviations: IF, immunofluorescence; WB, western blot.

### 2.4 Real‐Time PCR

Total RNA was isolated from podocytes using RNeasy Plus Mini Kit (Catalog No. 74134; Qiagen) with consecutive DNA digestion. RNA concentration and purity were assessed on the NanoDrop device (Thermofisher Scientific). After the isolation, reverse transcription was performed. Complementary DNA was subjected to real‐time PCR using LightCycler 480 (Roche), which employs gene‐specific primers and fluorescent hydrolysis probes (Table 2; Merck). To establish relative quantification of messenger RNA (mRNA) transcripts, the ΔΔC_t_ method was used with β‐actin serving as the reference gene.

**Table 2:** Sequences of primers and fluorescent probes for real‐time PCR analysis.

Protein	Gene	Accession no. for mRNA sequence	Primer sequence	Probe sequence	Product length (base pairs)
NOD2	*NOD2*	NM_001293557	Forward: GAACGTCATGCTAGAAGA	CTCTGCCTGGAGGAGAACCATC	115
			Reverse: GGACAACTTCAGGATTTTC		
Neutrophil elastase	*ELANE*	NM_001972.3	Forward: GTGGCGAATGTAAACGTC	CCTGGGAGCCCATAACCTCT	153
			Reverse: CTGGAGAATCACGATGTC		
Cathepsin G	*CTSG*	NM_001911.2	Forward: CCACAATATCCAGAGACG	AAACACCCAGCAACACATCACT	120
			Reverse: CTGCTCAGCTGCAATAAC		
Proteinase 3	*PRTN3*	NM_002777.3	Foward: CAGGAGCTCAATGTCACC	TGGTCACCTTCTTCTGCCGG	98
			Reverse: GAGTCTCCGAAGCAGATG		
β‐actin	*ACTB*	NM_001101.5	Forward: ATTGGCAATGAGCGGTTC	AGGCACTCTTCCAGCCTTC	76
			Reverse: GGATGCCACAGGACTCCA		

### 2.5 Immunofluorescence

Podocytes were cultured on a glass‐bottom dish (Catalog No. 81158; Ibidi). Cells were fixed in 4% formaldehyde for 20 min at room temperature with subsequent permeabilization in 0.1% Triton X‐100. Next, blocking was performed in a buffer containing 2% FBS, 2% bovine serum albumin, and 0.2% fish gelatin. Podocytes were then incubated with primary antibodies (Table 1) at 4°C overnight. On the next day, primary antibodies were washed off and cells were incubated with secondary antibodies (Catalog No. A11030; Thermofisher Scientific) diluted 1:200 in blocking solution for 2 h at room temperature. Parallel staining of the actin cytoskeleton was achieved with phalloidin (Catalog No. A12379; Thermofisher Scientific). Fluorescence analysis was conducted using a confocal microscope (Eclipse Ti; Nikon Instruments; RCM device, Confocal. nl).

### 2.6 ELISA

The ELISA assay for human NOD2 (Catalog No. EH10600; FineTest) was performed according to the manufacturer's instructions provided with the kit. Cell lysates were generated using RIPA buffer. An equal amount of protein was loaded into each well. Samples were diluted 10x.

### 2.7 Albumin Permeability Through the Podocyte Monolayer

To assess albumin permeability, the diffusion of FITC‐labeled BSA over a podocyte monolayer was measured as described previously (Audzeyenka et al. 2020; Piwkowska et al. 2012). Podocytes were cultured on inserts coated with type IV collagen (Catalog No. 354541; Corning). 2 h before the experiment, culture medium was replaced with 0.3 ml fresh serum‐free medium (SFM) in the upper compartment. The lower compartment contained 1.35 ml SFM supplemented with 1 mg/ml FITC‐albumin. After 1 h, 200 µl of the upper compartment SFM was relocated to a 96‐well plate. The concentration of FITC‐albumin was calculated by measurement of the absorbance at 490 nm using EnSpire Multilabel Reader 2300 (PerkinElmer).

### 2.8 NADPH Oxidase Activity

As previously mentioned (Piwkowska et al. 2010), lucigenin‐enhanced chemiluminescence was evaluated to evaluate NADPH oxidase activity using a Sirius 2 luminometer (Berthold Technologies). Applying a previously defined standard curve as a reference, the technique involved integrating the area under the curve to calculate the amount of superoxide formation (Münzel et al. 1996).

### 2.9 Protease Activity Assay

A fluorescent substrate was used to quantify the active form of NE (Jablaoui et al. 2020). A total of 20 µl of the substrate ABZ‐Met‐Pro‐Val‐Ala‐Trp‐Glu‐Tyr(3‐NO_2_)‐NH_2_ (final concentration in the test well: 47 µM) was incubated at 37°C with 10 µl of the sample—either cell lysate or extracellular medium, which was subjected to ultrafiltration using a 10 kDa Amicon Ultra Centrifugal Filter (Catalog No. UFC5010, Merck). The sample was diluted in 168 µl of assay buffer (100 mM Tris, 500 mM NaCl, pH 7.5) and 2 µl of DMSO. Before the experiment, samples were pre‐incubated for 30 min at room temperature in the aforementioned buffer with 2 µl of Sivelestat, a selective NE inhibitor (K. Li et al. 2024) dissolved in DMSO, resulting in a final concentration of 23 µM. This served as a specific activity control. The alteration in fluorescence intensity was monitored for 3 h (λ_Ex/Em_ = 320/450 nm).

To assess the biological activity of PR 3, a fluorescent substrate with the sequence ABZ‐Tyr‐Tyr‐Abu‐Asn‐Glu‐Pro‐Tyr(3‐NO_2_)‐NH_2_ was used (Popow‐Stellmaszyk et al. 2013). Each well received 155 µl of the aforementioned assay buffer, 15 µl of ddH₂O, and 10 µl of either cell lysate or extracellular medium, which was ultrafiltered as described above. The sample was pre‐incubated for 30 min at room temperature with 15 µl of Aprotinin, a serine protease inhibitor, in the same buffer (Zhirnov et al. 2011). Aprotinin was dissolved in H₂O, achieving a final concentration of 0.29 mM. Subsequently, 20 µl of the fluorescent substrate was added to each well (final concentration in the test well: 47 µM), and fluorescence was quantified for 3 h at 37°C (λ_Ex/Em_ = 320/450 nm). All measurements were conducted using a CLARIOStar microplate reader (BMG Labtech) and a Nunc F96 MicroWell Black Polystyrene Plate (Thermo Fisher, Catalog No. 237107).

### 2.10 Statistical Analysis

Data are presented as mean ± SEM. Normality of data distribution was checked using the Shapiro‐Wilk test. When comparing 2 groups of data sets parametric *t*‐test was used. When more than two groups of data were compared, parametric ANOVA was employed with an appropriate post‐hoc test. The analysis of the obtained data was performed in Prism 8.4.3 software (GraphPad).

## 3 Results

### 3.1 Podocytes Express Neutrophil‐Specific Serine Proteases

**Figure 1:**
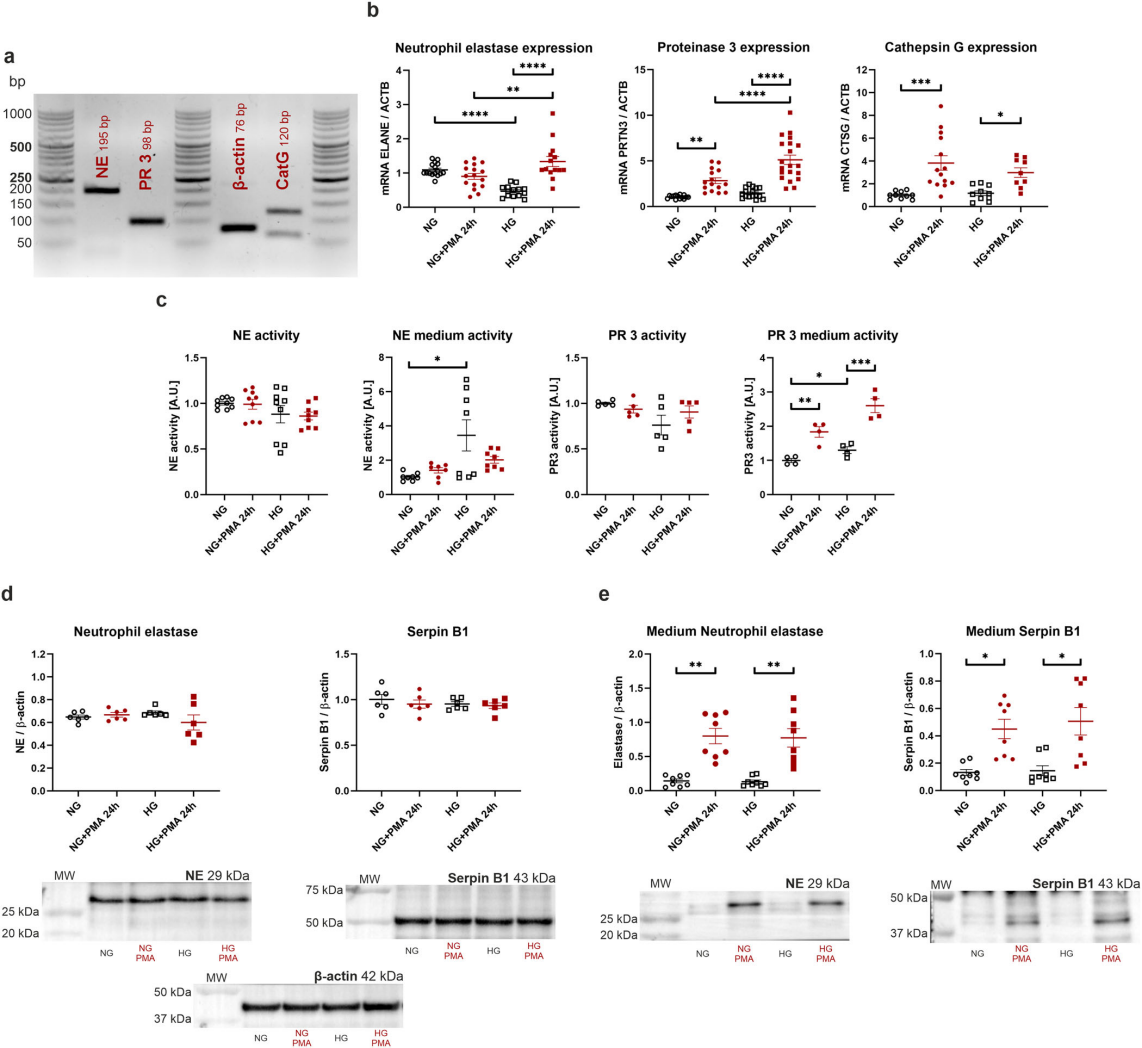
Podocytes exhibit the expression of neutrophil serine protease genes and possess the ability to produce functional proteins. (a) 2.5% agarose gel, which presents specific bands of real‐time PCR products of NE, PR 3, and CatG amplification. (b) Changes in the expression of ELANE (**p < 0.01, ****p < 0.0001, Sidak's multiple comparisons test, *n* = 14–16), PRTN3 (**p < 0.01, ****p < 0.0001, Sidak's multiple comparisons test, *n* = 14–20), and CSTG (*p < 0.05, ***p < 0.001, Sidak's multiple comparisons test, *n* = 9–14) after PMA stimulation. (c) The activity of serine proteases varies in culture medium harvested from the cells. NE activity was elevated in HG medium (*p < 0.05, Dunn's multiple comparisons test, *n* = 7–8), whereas PR 3 activity increased in all tested conditions (*p < 0.05, **p < 0.01, ***p < 0.001, Sidak's multiple comparisons test, *n* = 4). (d) No changes observed in NE and Serpin B1 amount. (e) Elevation in the amount of NE and Serpin B1 in culture medium after PMA addition (*p < 0.05, **p < 0.01, Dunn's multiple comparisons test, *n* = 8).

During our research, we established that podocytes were capable of expressing neutrophil‐specific serine proteases at the level of genes and functional proteins. Figure 1a presents specific products of real‐time PCR analysis of *ELANE*, *PRTN3*, and *CTSG*, which encode neutrophil elastase (NE), proteinase 3 (PR 3), and cathepsin G (CatG), respectively. It was also observed that expression of the above‐mentioned genes was dependent on the exposure of podocytes to PMA as well as high glucose (HG) in the culture environment. HG incubation resulted in over 50% reduction of *ELANE* expression. Addition of PMA to the HG medium caused almost twofold elevation in *ELANE* expression. The effect of PMA incubation was more profoundly marked in HG medium compared to NG (NG: 1.01 ± 0.05, NG + PMA 24 h: 0.90 ± 0.09, HG: 0.47 ± 0.04, HG + PMA 24 h: 1.34 ± 0.15; Figure 1b). *PRTN3* expression was increased after PMA addition, both in NG and HG medium, whereas the elevation was more robust in HG conditions than in NG at 248% and 154%, respectively. It is also worth noting that PMA supplementation resulted in greater *PRTN3* overexpression in HG than NG (NG: 1.05 ± 0.05, NG + PMA 24 h: 0.2.83 ± 0.31, HG: 1.48 ± 0.14, HG + PMA 24 h: 5.12 ± 0.51; Figure 1b). Lastly, *CTSG* expression was elevated after PMA stimulation in both NG (almost threefold) and HG (1.5‐fold) conditions (NG: 1.00 ± 0.10, NG + PMA 24 h: 3.81 ± 0.63, HG: 1.17 ± 0.21, HG + PMA 24 h: 2.98 ± 0.42; Figure 1b). Next, it was noticed that both NE and PR 3 activity increased in HG culture medium. NE activity elevated almost threefold in HG (NG: 1.03 ± 0.08, HG: 3.45 ± 0.91; Figure 1c), whereas PR 3 activity was also raised after PMA treatment (NG: 1.00 ± 0.06, NG + PMA 24 h: 1.83 ± 0.16, HG: 1.30 ± 0.10, HG + PMA 24 h: 0.41 ± 0.20; Figure 1c).

Due to the fact that there exist reports on the relationship between NOD2 activation and NE functions (Alyami et al. 2019), the focus was placed on the description of NE and its specific inhibitor, Serpin B1, in the studied conditions. No changes were observed in the protein amount of both NE and Serpin B1 (Figure 1d); however, PMA incubation caused severe secretion of analyzed proteins into the culture medium. Almost fivefold increase in medium NE amount was established both in NG and HG (NG: 0.14 ± 0.03, NG + PMA 24 h: 0.80 ± 0.11, HG: 0.13 ± 0.03, HG + PMA 24 h: 0.77 ± 0.13; Figure 1e). We observed the same tendency regarding Serpin B1, with 240% increase after PMA addition (NG: 0.13 ± 0.02, NG + PMA 24 h: 0.45 ± 0.07, HG: 0.15 ± 0.04, HG + PMA 24 h: 0.51 ± 0.10; Figure 1e).

It was established that podocytes expressed NSPs and produced functional proteins whose extracellular activity depended on inflammatory induction. Along with NE release, its inhibitor, Serpin B1, was also secreted.

### 3.2 Podocytes Express NOD2 Protein Which Varies in Hyperglycemic Conditions

**Figure 2:**
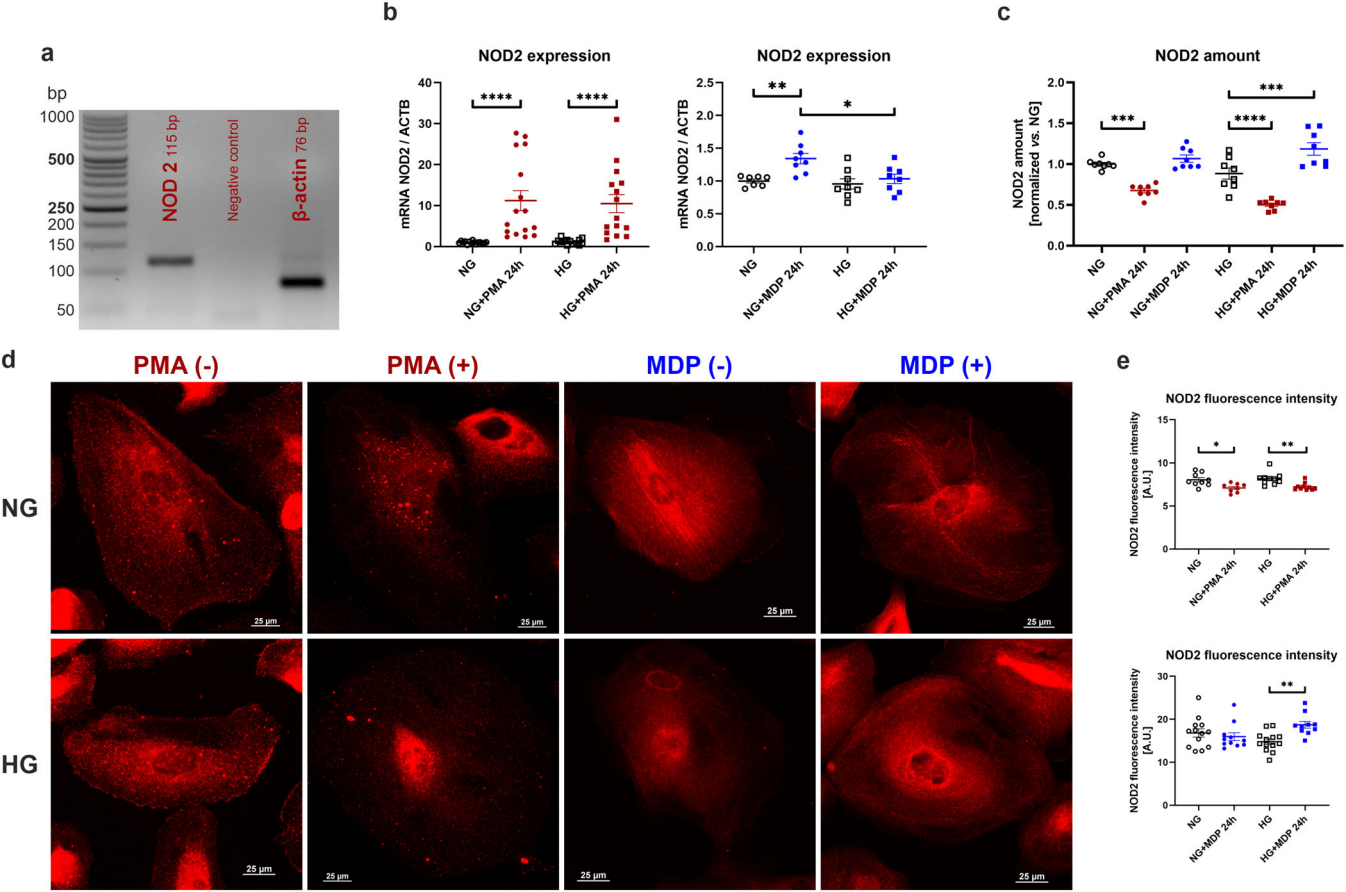
Podocytes express NOD2 protein, which can be stimulated by MDP. (a) 2.5% agarose gel presents a specific product of NOD2 amplification. (b) PMA causes increase in NOD2 expression (****p < 0.0001, Dunn's multiple comparisons test, *n* = 14–16), while MDP stimulates NOD2 amplification only in NG conditions (*p < 0.05, **p < 0.01, Sidak's multiple comparisons test, *n* = 7–8). (c) The amount of NOD2 decreases after PMA stimulation, but elevates after the addition of MDP to HG medium (***p < 0.001, ****p < 0.0001, Sidak's multiple comparisons test, *n* = 8). (d) Confocal images of NOD2 staining (scale bar is 25 µm). (e) Fluorescence intensity of NOD2 stained cells after stimulation with PMA (*p < 0.05, **p < 0.01, Dunn's multiple comparisons test, *n* = 8), and MDP (**p < 0.01, Dunn's multiple comparisons test, *n* = 8–10).

In the next part of the research, the expression of *NOD2* gene encoding NOD2 protein was evaluated in podocytes. Figure 2a presents real‐time PCR product of *NOD2* gene amplification. It was observed that PMA stimulation caused 10‐fold increase in *NOD2* expression, both in NG and HG (NG: 1.00 ± 0.31, NG + PMA 24 h: 11.21 ± 2.44, HG: 1.19 ± 0.18, HG + PMA 24 h: 10.50 ± 2.20; Figure 2b). Interestingly, after MDP addition, which is a specific NOD2 activator, an elevation in *NOD2* expression in NG conditions was noted (NG: 1.00 ± 0.3, NG + MDP 24 h: 1.34 ± 0.08, HG: 0.96 ± 0.08, HG + MDP 24 h: 1.03 ± 0.07; Figure 2b). An opposite effect was observed in the case of NOD2 protein. Stimulation with PMA resulted in a reduction of NOD2 levels by more than 30% under both NG and HG conditions. In contrast, activation of NOD2 by MDP led to an increase in protein levels exclusively under HG conditions (NG: 1.00 ± 0.02, NG + PMA 24 h: 0.68 ± 0.03, NG + MDP 24 h: 1.07 ± 0.04, HG: 0.88 ± 0.07, HG + PMA 24 h: 0.50 ± 0.02, HG + MDP 24 h: 1.17 ± 0.08; Figure 2c). To verify the above‐mentioned findings, the immunofluorescence staining of NOD2 protein was performed (Figure 2d), which confirmed earlier observations. Fluorescence intensity was reduced after PMA addition (NG: 8.05 ± 0.25, NG + PMA 24 h: 7.11 ± 0.18, HG: 8.18 ± 0.22, HG + PMA 24 h: 7.24 ± 0.14; Figure 2e), but was increased after MDP stimulation (HG: 14.80 ± 0.63, HG + MDP 24 h: 18.72 ± 0.79; Figure 2e). It is noteworthy that following PMA stimulation, in addition to a decrease in fluorescence intensity, the distribution of NOD2 also changed. Prominent granules could be observed in the perinuclear region, unlike the uniform distribution seen under control conditions. The observed granulation was the result of the well‐characterized action of PMA itself (Giambelluca et al. 2022; Mukherjee et al. 2023). In contrast, stimulation of podocytes with MDP did not significantly alter the distribution of NOD2, which exhibited a diffuse staining pattern.

It was noted that podocytes produced NOD2, whose levels decreased after PMA stimulation, whereas specific activation by MDP increased NOD2 abundance in insulin‐insensitive cells.

### 3.3 MDP Stimulation Causes Increase in NSP Expression and Activity in Podocytes

**Figure 3:**
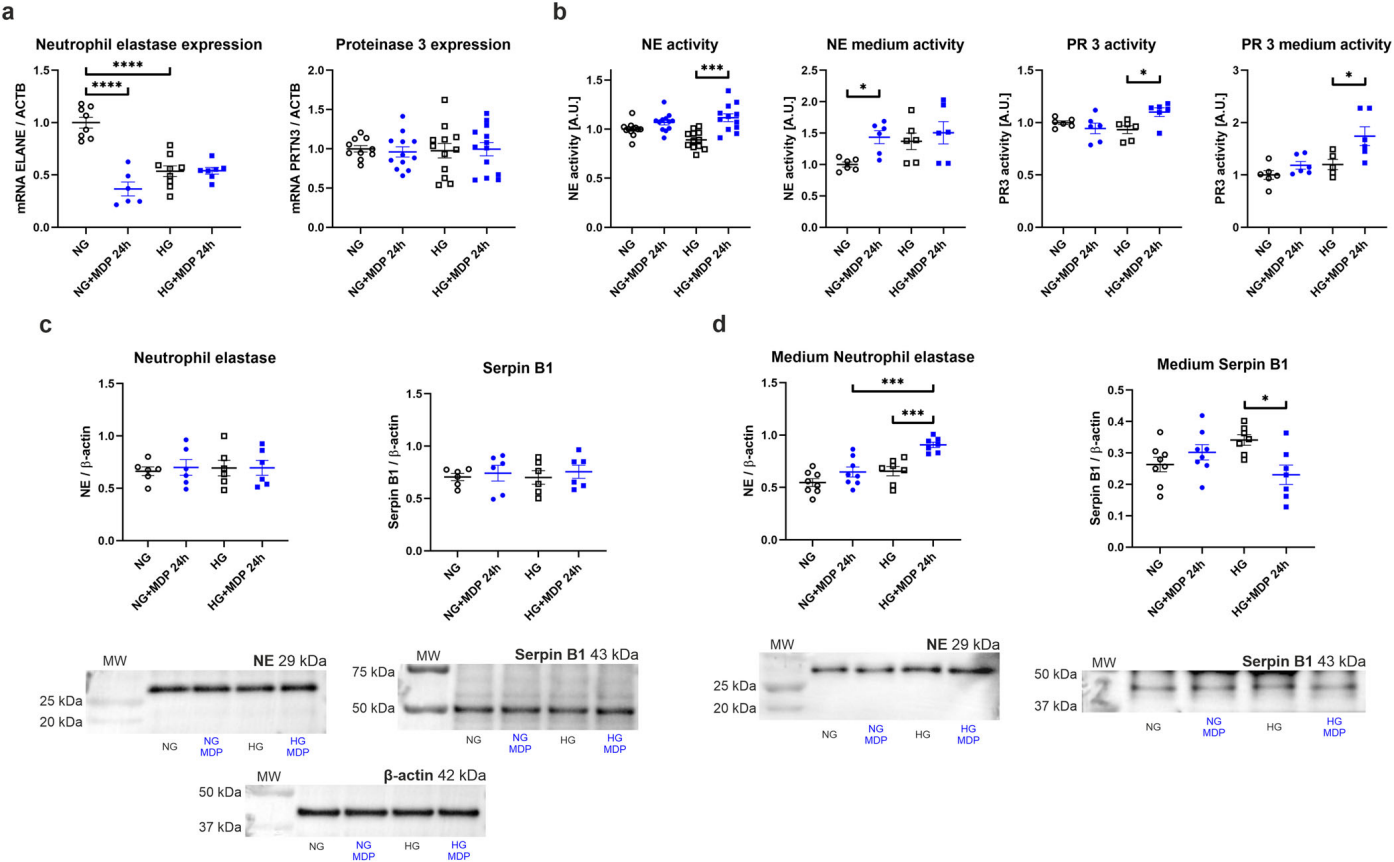
NOD2 activation influences NSPs expression and activity with subsequent increase in NE and PR 3 secretion. (a) MDP treatment leads to decrease in ELANE expression only in podocytes cultured in NG medium. HG conditions cause reduction in ELANE mRNA amount compared to NG (****p < 0.0001, Sidak's multiple comparisons test, *n* = 6–9). No alterations are observed regarding PRTN3 expression. (b) NOD2 activation leads to an increase in intracellular NE activity in HG conditions (***p < 0.001, Dunn's multiple comparisons test, *n* = 12), as well as in NG medium (*p < 0.05, Sidak's multiple comparisons test, *n* = 6). PR 3 activity rises in HG conditions after MDP addition, both in cell lysates (*p < 0.05, Dunn's multiple comparisons test, *n* = 6) and harvested medium (*p < 0.05, Sidak's multiple comparisons test, *n* = 5–6). (c) No alterations observed regarding NE and Serpin B1 protein level after MDP treatment. (d) Elevation in NE secretion after NOD2 activation in HG medium (***p < 0.001, Sidak's multiple comparisons test, *n* = 7–8), which is more profoundly noted than in NG conditions (***p < 0.001, Sidak's multiple comparisons test, *n* = 7–8). Increased secretion of NE under HG conditions was accompanied by a significant decrease in the Serpin B1 amount, the most potent NE inhibitor (*p < 0.05, Sidak's multiple comparisons test, *n* = 7–8).

Given that podocytes express the NOD2 protein, the aim was to investigate how its activation affects the expression and activity of NSPs. It was noted that NOD2 activation causes 63% reduction in *ELANE* expression in NG conditions, but MDP stimulation failed to influence the amount of *ELANE* transcript in HG medium. The reduction by half in *ELANE* expression after HG treatment compared to NG was observed, as presented in Section 3.1 (NG: 1.00 ± 0.05, NG + MDP 24 h: 0.37 ± 0.07, HG: 0.54 ± 0.05, HG + MDP 24 h: 0.54 ± 0.03; Figure 3a). Conversely, the *PRTN3* mRNA level was not changed in the analyzed conditions (Figure 3a). In the next step, it was established that activation of NOD2 by MDP resulted in 26% elevation in NE activity in HG conditions (HG: 0.89 ± 0.03, HG + MDP 24 h: 1.12 ± 0.04; Figure 3b) and 44% in NG culture medium (NG: 1.00 ± 0.04, NG + MDP 24 h: 1.44 ± 0.10; Figure 3b). NOD2 activation led to an 18% increase in PR 3 activity in HG conditions (HG: 0.93 ± 0.04, HG + MDP 24 h: 1.10 ± 0.04; Figure 3b) and, analogously, a 45% increment in HG culture medium (HG: 1.20 ± 0.10, HG + MDP 24 h: 1.74 ± 0.18; Figure 3B). No changes were observed in the amount of NE and Serpin B1 protein after NOD2 activation (Figure 3c). However, the amount of analyzed proteins varied in the culture medium harvested from the cells. NE quantity was elevated almost 40% in HG medium after MDP stimulation, and this increase was also at the level of 40% compared to NG medium with MDP addition (NG + MDP 24 h: 0.65 ± 0.05, HG: 0.66 ± 0.05, HG + MDP 24 h: 0.91 ± 0.03; Figure 3d). Moreover, a significant 32% reduction of Serpin B1 in HG medium after MDP‐dependent NOD2 activation was noticed (HG: 0.34 ± 0.02, HG + MDP 24 h: 0.23 ± 0.03; Figure 3d).

NSP activity was shown to depend not only on PMA but also on MDP incubation. Upon NOD2 activation, extracellular Serpin B1 levels decreased, suggesting a loss of its ability to inhibit NE. These findings indicated that NOD2 activation shifted the balance between NE and Serpin B1.

### 3.4 PMA and MDP Alter Podocyte Cytoskeleton and Albumin Permeability

**Figure 4:**
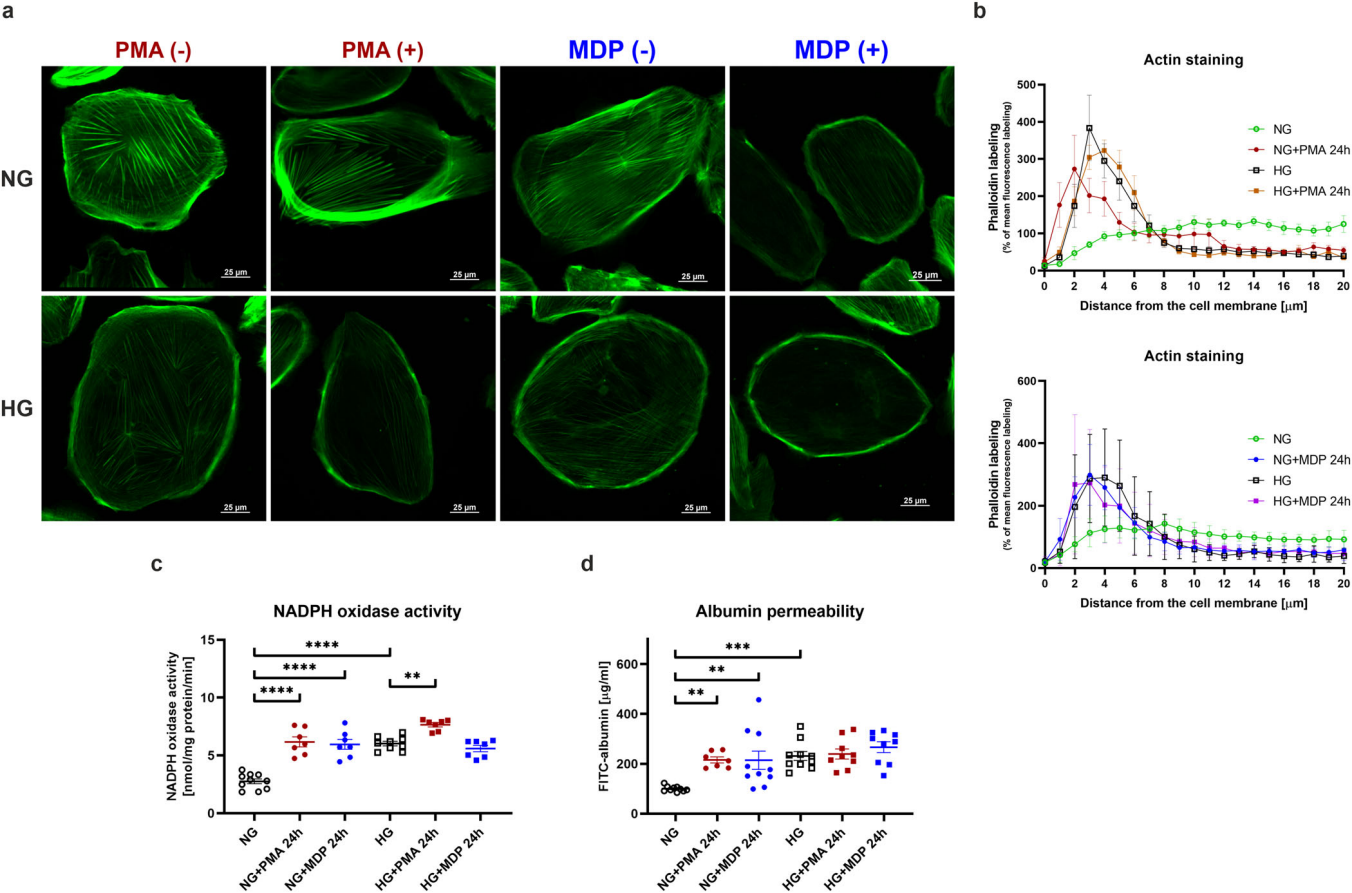
PMA and MDP disrupts delicate actin architecture both in NG and HG conditions. (a) Representative images of phalloidin staining showing distinct perimembranous distribution of actin fibers after treatment of podocytes with PMA and MDP in NG or HG conditions (scale bar is 25 µm). (b) Quantitative representation of the changes observed in section (A) (for PMA *n* = 7–9, for MDP *n* = 10–12). (c) After the induction or inflammation or NOD2 activation the activity of NADPH oxidase raises, similarly to incubation of podocytes in HG medium (**p < 0.01, ****p < 0.0001, Sidak's multiple comparisons test, *n* = 7–10). (d) One of the consequences of actin cytoskeleton rearrangement and increased oxidative stress is the enhanced permeability of the podocyte monolayer to albumin. PMA and MDP induced disruption of cell junction integrity in podocyte cultures maintained in HG medium; however, no cumulative effect was observed when these compounds were combined with HG conditions (**p < 0.01, ***p < 0.001, Sidak's multiple comparisons test, *n* = 7–10).

In the subsequent phase of the study, the aim was to investigate whether and in what manner the aforementioned changes influence podocyte functions. Firstly, phalloidin staining of the actin cytoskeleton in tested conditions was performed (Figure 4a). Both PMA and MDP caused the rearrangement of actin distribution throughout the cell. Actin fibers were translocated to the periphery of podocyte′s cell membrane, which was a similar image to that obtained under HG incubation (Figure 4b). Next, the oxidative balance of podocytes was investigated, as it had been demonstrated that its disruption affected, among other factors, the distribution of the actin cytoskeleton (Liu et al. 2013). The activity of NADPH oxidase, one of the main generators of reactive oxygen species (ROS) in podocytes, was examined (Piwkowska et al. 2010, 2013). The obtained findings revealed that, under control conditions, PMA induced an almost 125% increase in NADPH oxidase activity, while MDP led to an elevation of over 115%. Both compounds resulted in a comparable growth in NADPH oxidase activity to that observed in high‐glucose medium alone. However, under hyperglycemic conditions, only PMA stimulation resulted in a statistically significant increase in ROS generation by NADPH oxidase, amounting to just 27% (NG: 2.76 ± 0.21 nmol/mg protein/min, NG + PMA 24 h: 6.17 ± 0.43 nmol/mg protein/min, NG + MDP 24 h: 5.96 ± 0.44 nmol/mg protein/min, HG: 6.03 ± 0.20 nmol/mg protein/min, HG + PMA 24 h: 7.65 ± 0.18 nmol/mg protein/min, HG + MDP 24 h: 5.59 ± 0.28 nmol/mg protein/min; Figure 4c). Modifications in the actin cytoskeleton distribution and the presence of oxidative stress contributed to the impairment of podocyte function in albumin retention. Analysis of albumin permeability across the podocyte monolayer revealed that PMA and the NOD2 activator led to disruption of intercellular junctions, as evidenced by the leakage of FITC‐labeled albumin into the upper compartment, mimicking the urinary space of Bowman′s capsule. The observed elevation in permeability (116% for PMA and 114% for MDP) was comparable to that seen under HG conditions (131%). Although, no additive effect of PMA or MDP was detected in podocytes cultured in HG medium, a 15% increasing trend was observed with the addition of MDP to HG medium. (NG: 99.88 ± 3.47 µg/ml, NG + PMA 24 h: 215.30 ± 12.03 µg/ml, NG + MDP 24 h: 214.10 ± 36.91 µg/ml, HG: 231.10 ± 18.05 µg/ml, HG + PMA 24 h: 239.10 ± 20.12 nmol/mg protein/min, HG + MDP 24 h: 266.60 ± 21.70 µg/ml; Figure 4d)

These results indicated that both PMA‐induced inflammation and NOD2 activation led to the rearrangement of actin filaments and dysregulation of the redox balance, which resulted in increased albumin permeability.

### 3.5 Development of NE‐Silenced Podocyte Cell Line

**Figure 5:**
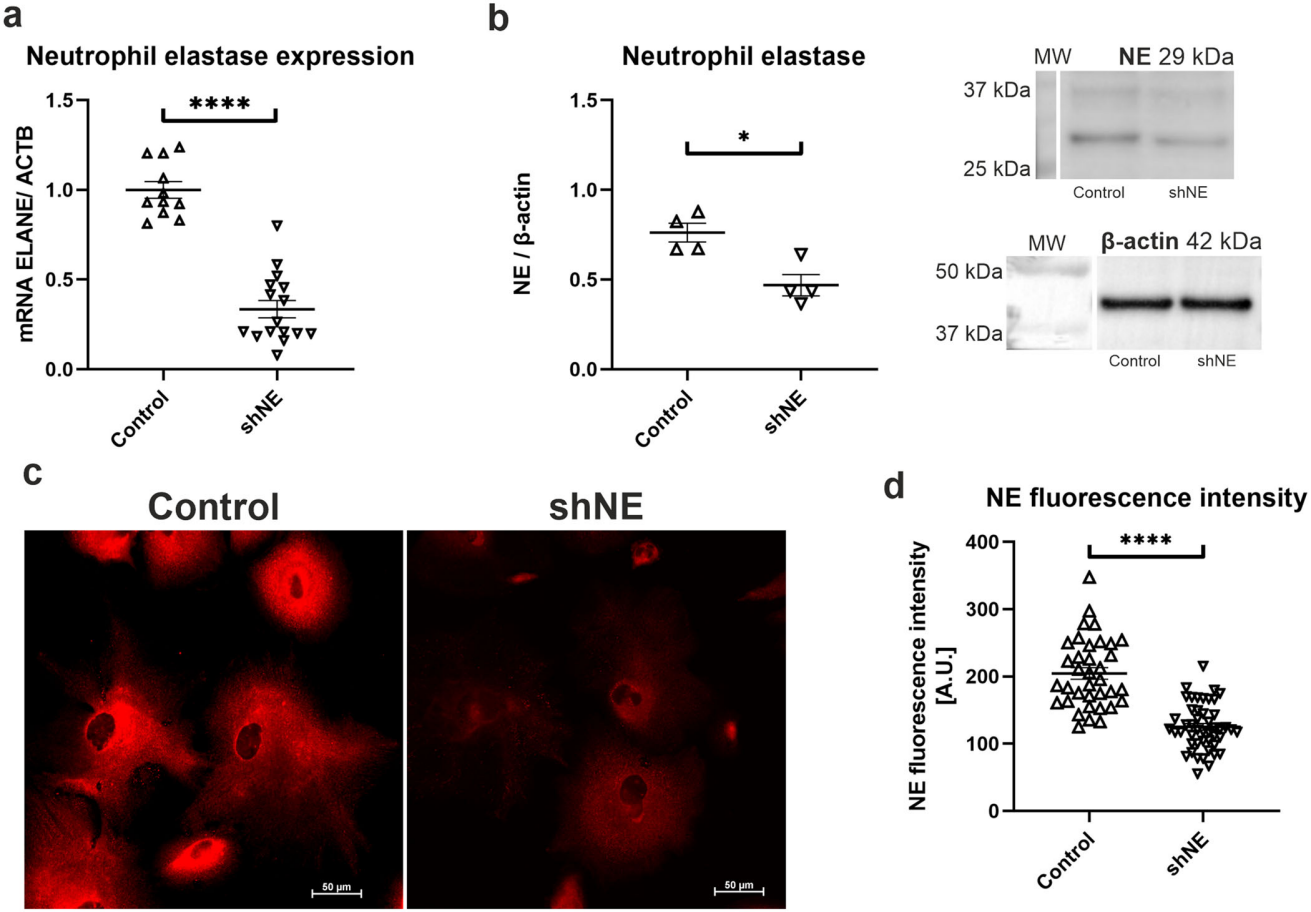
Generation of podocyte cell line with silenced ELANE gene. (a) Real‐time PCR analysis showed a reduction in ELANE expression in lentiviral‐transduced podocytes (****p < 0.0001, unpaired *t* test, *n* = 11–16). (b) Western blot assay presented reduced amount of NE protein in shNE podocytes (*p < 0.05, unpaired *t* test, *n* = 4). (c) Confocal images comparing fluorescence of Control cells and shNE podocytes, indicating that the latter generate a significantly weaker signal (scale bar is 50 µm). (d) Quantitative analysis of NE fluorescence intensity in control cells and NE‐silenced cells (****p < 0.0001, unpaired *t* test, *n* = 36–47).

To further assess the relationship between NOD2 activation and NSPs mobilization, a podocyte cell line with silenced *ELANE* gene by lentiviral transduction was generated. Real‐time PCR analysis revealed that downregulation of *ELANE* gene was successful to an extent of 67% (Control: 1.00 ± 0.05, shNE: 0.33 ± 0.05; Figure 5a). At the protein level, *ELANE* silencing resulted in 38% decrease in NE amount (Control: 0.76 ± 0.05, shNE: 0.47 ± 0.06; Figure 5b). Immunofluorescent staining of NE was implemented to verify the above‐mentioned findings (Figure 5c), which established 39% decrease of fluorescence intensity in shNE podocytes (Control: 204.6 ± 8.62, shNE: 125.2 ± 5.03; Figure 5d).

### 3.6 Downregulation of NE Improves Podocyte Function

**Figure 6:**
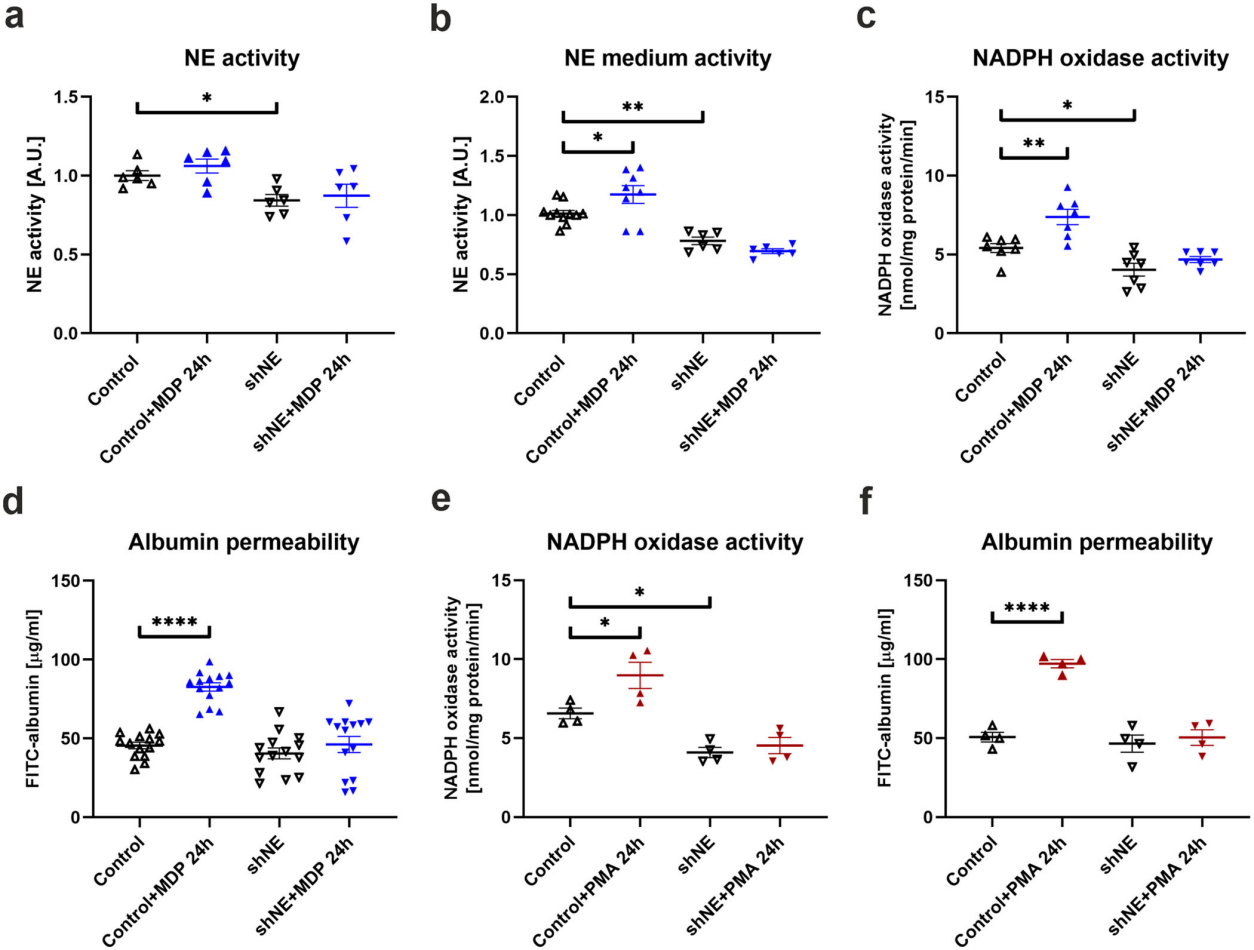
Silencing of NE mitigated oxidative stress and prevented the impairment of albumin filtration in podocytes following MDP stimulation as well as PMA treatment. (a) NE activity decreased in NE downregulated cells (*p < 0.05, Sidak's multiple comparisons test, *n* = 6). (b) NE activity in the culture medium harvested from shNE cells was significantly reduced than in control cells, and MDP stimulation failed to induce the release of enzymatically active NE. (*p < 0.05, **p < 0.01, Sidak's multiple comparisons test, *n* = 6–11). (c) MDP treatment did not trigger oxidative stress in podocytes with downregulated NE, as evidenced by the absence of an elevation in NADPH oxidase activity (*p < 0.05, **p < 0.01, Sidak's multiple comparisons test, *n* = 7). (d) Silencing of NE improved podocyte function, as stimulation of these cells with MDP did not cause elevation in albumin permeability ****p < 0.0001, Dunn's multiple comparisons test, *n* = 14). (e) Similar to the observations with MDP, shNE podocytes stimulated with PMA did not show increased NADPH oxidase activity, while silencing alone resulted in its reduction (*p < 0.05, Sidak's multiple comparisons test, *n* = 4). (f) PMA treatment did not increase albumin permeability in shNE cells, as observed in control podocytes (****p < 0.0001, Sidak's multiple comparisons test, *n* = 4).

In the next stage of the study, the effect of *ELANE* gene silencing on elastase activity and redox balance in podocytes was investigated. The 16% reduction of NE activity was noted in shNE cells (Control: 1.00 ± 0.03, shNE: 0.84 ± 0.04; Figure 6a). It was also found that in the medium collected from control cells stimulated with MDP, NE activity was elevated, similarly to the genetically unmodified cells described in Section 3.3. Additionally, a 23% reduction in extracellular activity was observed in shNE cells, and MDP stimulation did not induce an increase in NE activity (Control: 1.01 ± 0.03, Control + MDP 24 h: 1.17 ± 0.08, shNE: 0.78 ± 0.03, shNE + MDP 24 h: 0.70 ± 0.02; Figure 6b). Based on NADPH oxidase activity analysis, downregulation of NE improved the redox state of podocytes. In Control cells, MDP stimulation caused almost 40% boost in ROS generation by NADPH oxidase, whereas in shNE podocytes we observed 25% significant decline in NADPH oxidase activity compared to Control cells. Furthermore, MDP treatment did not influenced oxidase activity in podocytes with depleted NE formation, which suggests that NE was somewhat involved in the process of podocyte injury, and this phenomenon was dependent on NOD2 activation (Control: 5.41 ± 0.29 nmol/mg protein/min, Control + MDP 24 h: 7.38 ± 0.49 nmol/mg protein/min, shNE: 4.04 ± 0.41 nmol/mg protein/min, shNE + MDP 24 h: 4.68 ± 0.19 nmol/mg protein/min; Figure 6c). Finally, an investigation was conducted to determine how the previously described alterations influenced the key physiological function of podocytes, which is preventing the passage of macromolecules into the ultrafiltrate. MDP treatment led to 82% increment in albumin permeability in Control cells, but failed to dysregulate podocyte function in shNE cells. Additionally, there was a 10% downward trend in albumin permeability between shNE podocytes and Control cells, (Control: 45.47 ± 2.05 µg/ml, Control + MDP 24 h: 82.68 ± 2.64 µg/ml, shNE: 40.47 ± 3.44 µg/ml, shNE + MDP 24 h: 46.23 ± 5.10 µg/ml; Figure 6d). Similar results were observed in silenced podocytes stimulated with PMA. Following NE downregulation, NADPH oxidase activity was reduced, and PMA treatment did not enhance its activity (Control: 6.57 ± 0.34 nmol/mg protein/min, Control + PMA 24 h: 8.98 ± 0.83 nmol/mg protein/min, shNE: 4.10 ± 0.32 nmol/mg protein/min, shNE + PMA 24 h: 4.53 ± 0.51 nmol/mg protein/min; Figure 6e). An identical relationship was established for albumin permeability in silenced podocytes. NE silencing rendered the cells insensitive to PMA, as reflected by albumin permeability levels nearly identical to those in Control cells (Control: 50.64 ± 3.08 µg/ml, Control + PMA 24 h: 97.20 ± 2.61 µg/ml, shNE: 46.50 ± 5.48 µg/ml, shNE + PMA 24 h: 50.41 ± 4.97 µg/ml; Figure 6f).

These data indicated that NE downregulation rendered podocytes insensitive to both PMA and MDP. In NE‐depleted cells, oxidative stress did not occur, and albumin permeability remained at levels comparable to control cells.

## 4 Discussion

**Figure 7:**
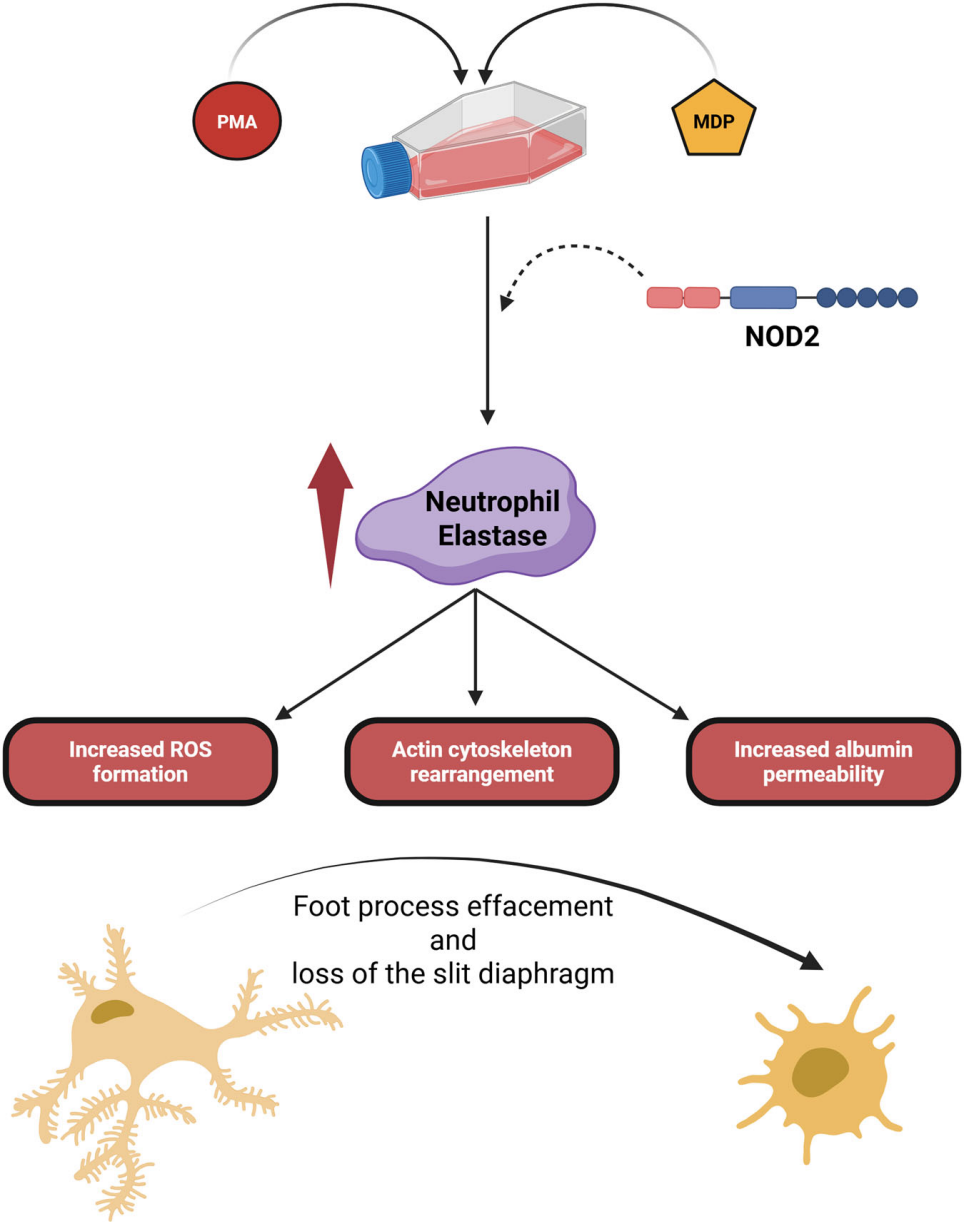
A schematic representation of the key findings. Induction of inflammation in podocytes by PMA or specific activation of NOD2 by MDP leads to NE‐mediated impairment of podocyte function. This is manifested by increased ROS generation via NADPH oxidase, reorganization of the actin cytoskeleton, and, consequently, increased podocyte permeability to albumin. The dashed arrow indicates the mechanism of NE activation by NOD2, which requires further detailed investigation.

This study provided evidence that podocytes are capable of expressing NSPs, which exhibit intracellular activity and are also secreted into the extracellular environment. It also demonstrated the similarity between podocytes and immune cells, as indicated by the presence of the intracellular NOD2 receptor, whose specific activation influences NSP activity and secretion. Furthermore, it was established that loss of insulin sensitivity induced by a 5‐day incubation with HG in podocytes led to an increased NOD2 level upon activation. Additionally, through *ELANE* gene silencing, it was determined that NE acts as an effector of NOD2‐mediated podocyte integrity disruption, resulting in increased albumin permeability and redox shift favoring ROS generation. A brief summary of the obtained findings is presented in Figure 7.

There is substantial evidence supporting the involvement of neutrophils in kidney damage (Davies et al. 1978; Johnson et al. 1987), with a particular emphasis on the role of NE (Kuźniar et al. 2007; Oda et al. 1997; Suzuki et al. 1998). Earlier research has linked both key neutrophil defense mechanisms—degranulation and ROS production—to filtration barrier impairment, primarily through GBM degradation (Davies et al. 1978; Schrijver et al. 1989). There is evidence suggesting the direct involvement of neutrophils in podocyte injury. Caster et al. demonstrated that inhibiting neutrophil′s granule exocytosis reduced the severity of proteinuria in a mouse model of glomerulonephritis. Moreover, in vitro stimulation of podocytes with granule content led to foot process effacement, resulting from disruptions in actin cytoskeleton delicate architecture (Caster et al. 2018). Stimulated podocytes were capable of releasing pro‐inflammatory cytokines and chemokines, which attracted neutrophils through chemotaxis, resulting in a positive feedback loop that exacerbated injury (Caster et al. 2018). NE may contribute to this damage as one of the key components of neutrophil granules. First, NE can activate the epidermal growth factor, which, as demonstrated by Bollée et al. participates in podocyte injury (Bollée et al. 2011). Second, in our study, we discovered that NOD2 activation increases both intracellular and extracellular activity of NE and PR 3. The link between NOD2 activation and NSPs function was previously established by Alyami et al (Alyami et al. 2019). It is therefore likely that NOD2 activation induces negative cytoskeletal rearrangement through intracellular NE activity, while also contributing to intercellular junction disruption, the combined effect of which is observed as an elevation in albumin permeability. It is also worth emphasizing that after MDP stimulation in HG medium, the amount of NE elevated while its inhibitor, Serpin B1, simultaneously decreased. This may suggest a reduced ability of Serpin B1 to downregulate NE in these conditions.

Serpin B1 exhibits its NSPs inhibitory capacity through a highly specific mechanism requiring irreversible covalent binding to the target protease, primarily NE, as well as to PR 3 (Jégot et al. 2011; Majewski et al. 2016; Potempa et al. 1994; Silverman et al. 2001). The phenomenon we observed may be attributed to inhibitor depletion effects. Serpin B1 also functions as a cytoprotective protein that safeguards cells against intracellular NE activity, thereby preventing autoproteolysis. Consequently, maintenance of steady‐state intracellular Serpin B1 levels seems to be essential for cellular homeostasis (Benarafa 2015; Benarafa et al. 2007; Burgener et al. 2019). It is plausible that NE release into the extracellular medium is not accompanied by proportionally enhanced Serpin B1 exocytosis, resulting in progressive depletion of the inhibitor pool and subsequent decline in its extracellular concentrations over time. It has also been established that Serpin B1 and NE can mediate insulin signaling and the development of insulin resistance. Studies have demonstrated that mice fed a high‐fat diet exhibit increased NE activity, which induces cellular insulin resistance, while NE deletion in obese mice results in reduced inflammation of insulin‐sensitive tissues (Gough 2012; Talukdar et al. 2012). This is accompanied by improved glucose tolerance, enhanced insulin sensitivity and prolonged insulin signaling (D'Souza et al. 2021; Talukdar et al. 2012). Conversely, Serpin B1 activity appears to function by sensitizing tissues to insulin action. Evidence exists for enhanced pancreatic β‐cell proliferation in response to Serpin B1 produced by hepatocytes (El Ouaamari et al. 2016). Clinical studies in patients have shown that serum Serpin B1 levels are positively correlated with the HOMA2‐%S index, which reflects the degree of insulin sensitivity, as well as with glycated hemoglobin (Hb_A1c_) (Kyohara et al. 2022; Takebayashi et al. 2016). Based on the aforementioned evidence, it is plausible that our observed increase in NSP activity and decrease in extracellular Serpin B1 levels may similarly contribute to the loss of podocyte insulin sensitivity.

It was found that NOD2 could potentially mediate the development of insulin resistance. In their study, Du et al. demonstrated that incubating mouse podocytes under HG conditions led to an increase in NOD2 protein levels (Du et al. 2013). Similarly, Wang et al. reached the same conclusion while working with human podocytes (Y. Wang et al. 2024). However, in the former study, incubation with high glucose lasted 24 h, while in the latter, it lasted 48 h, in contrast to our 5‐day culture period. This extended incubation time was not chosen arbitrarily. According to our previous research, a 5‐day incubation of podocytes in HG leads to loss of insulin sensitivity, manifested as reduced intracellular glucose uptake via the glucose transporter type 4 (GLUT4) transporter (Rogacka et al. 2014). In our experimental model, we did not observe any changes in NOD2 levels when podocytes lost insulin sensitivity. Moreover, NOD2 protein amount decreased following inflammation induction with PMA. This phenomenon may be attributed to heat shock protein 90 (HSP90) dissociation and rapid ubiquitination of NOD2 following its activation, leading to its subsequent proteasomal degradation (K. H. Lee et al. 2012). Furthermore, in lung cancer cells, the suppressor of cytokine signaling 3 (SOCS3) mediates in NOD2 ubiquitination, which results in proteasomal decomposition (Jeong et al. 2024). A compensatory mechanism that counteracts this process could be the elevated *NOD2* transcript level following PMA stimulation. The increased degradation of NOD2 following PMA stimulation can be explained through two potential mechanisms. In colonic epithelial cells, it has been demonstrated that PMA stimulation leads to the dissociation of HSP90 from inhibitor of NF‐κB kinase (IKK) (Park et al. 2007). HSP90 is a chaperone protein that stabilizes its interacting partners, thereby prolonging their half‐life. The detachment of HSP90 from IKK resulted in the proteasomal degradation of the latter. A similar mechanism may occur in podocytes, which, like colonic epithelial cells, are also epithelial in nature. A second explanation could be that PMA is known to induce the upregulation of SOCS3 (E. B. Lee et al. 2010; Terstegen et al. 2000). There are evidence, which indicates that podocytes express SOCS3 (Fukuta et al. 2021; Kuravi et al. 2014). Therefore, stimulation of podocytes by PMA may lead to the recruitment of SOCS3, which participates in the ubiquitination of NOD2, thereby contributing to the observed reduction in its levels.

Interestingly, in insulin‐insensitive podocytes, specific activation of NOD2 may not lead to its degradation in proteasomes, as indicated by the increased protein amount under these conditions, despite the absence of changes in *NOD2* gene expression. The role of NOD2 in the development of insulin resistance appears to be counterintuitive. In vivo studies have reported that NOD2 stimulation by MDP improves insulin sensitivity, enhances glucose tolerance, and prevents obesity (Cavallari et al. 2017; Denou et al. 2015; Williams et al. 2020). However, other studies suggest that NOD2 activation contributes to insulin resistance and impaired glucose tolerance, particularly in muscle cells (Maurya et al. 2015; Tamrakar et al. 2010). Additionally, the earlier‐mentioned research indicates that short‐term stimulation of podocytes with MDP deregulates their insulin response (Du et al. 2013). Notably, in both myocytes and podocytes, glucose uptake is mediated by the insulin‐dependent transporter GLUT4. It therefore appears that the role of NOD2 in the development of insulin resistance and other metabolic disorders is highly tissue‐specific. In this study, we focused on investigating the relationship and interplay between active NOD2 and NSPs in both control and insulin‐insensitive podocytes, as insulin resistance is the most common cause of podocyte dysfunction (Artunc et al. 2016; Coward and Fornoni 2015; Rogacka 2021). Based on our collected data, it is difficult to draw definitive conclusions regarding the involvement of NOD2 in the development of podocyte insulin resistance, which represents a limitation of our research and requires further studies. We acknowledge that a limitation of the present study is also the selective silencing of NE without concomitant targeting of other NSPs. Nevertheless, this choice is strongly supported by existing literature, which highlights NE as a principal mediator of extracellular matrix remodeling—a process central to the pathogenesis of various diseases, including glomerulopathies (Chua 2006; Garratt et al. 2015; Oda et al. 1997). We are also aware of certain discrepancies between the gene expression of NSPs and their protein abundance or enzymatic activities. NSPs are highly specific enzymes whose production in neutrophils occurs at precursor stages—primarily the myeloblast and promyelocyte. In mature neutrophils, NSPs are stored in granules, and their expression decreases (Benarafa and Simon 2017; Othman et al. 2022). The ability to synthesize and secrete NSPs is a novel phenomenon that we have observed in podocytes; therefore, the characterization of these proteases in podocytes requires further investigation.

In summary, in this study, we demonstrated that podocytes synthesize active forms of neutrophil serine proteases—neutrophil elastase, proteinase 3, and cathepsin G—that exhibit activity both intracellularly and are secreted into the culture medium. The activity of these NSPs contributed to the dysregulation of the filtration function of podocytes, particularly when the cells lose sensitivity to insulin, a hormone crucial to their physiology. We also established that activation of NOD2 seems to play a role in the recruitment of NSPs. However, the mechanism by which NOD2 influences NSPs and the development of insulin resistance requires further detailed investigation.

## Author Contributions

Tomasz Kulesza prepared the original draft of the manuscript. Agnieszka Piwkowska designed the study. Tomasz Kulesza, Aleksandra Wróblewska, Patrycja Rachubik, Irena Audzeyenka, Dorota Rogacka, Agnieszka Piwkowska, Wiktoria Mallek, Magdalena Wysocka, and Justyna Sawicka performed the experiments and analyzed the data. Adam Lesner provided the methods. Agnieszka Piwkowska, Dorota Rogacka, and Irena Audzeyenka revised the manuscript. Agnieszka Piwkowska obtained funding and managed the project. All authors approved the final version of the manuscript.

## Conflicts of Interest

The authors declare no conflicts of interest.

## Data Availability

The data that support the findings of this study are available from the corresponding author upon reasonable request.

## References

[jcp70116-bib-0001] Alyami, H. M. , L. S. Finoti , H. S. Teixeira , A. Aljefri , D. F. Kinane , and M. R. Benakanakere . 2019. “Role of NOD1/NOD2 Receptors in Fusobacterium Nucleatum Mediated NETosis.” Microbial Pathogenesis 131: 53–64. 10.1016/j.micpath.2019.03.036. 30940608

[jcp70116-bib-0002] Ambrus, L. , A. Oláh , T. Oláh , et al. 2015. “Inhibition of TRPC6 by Protein Kinase C Isoforms in Cultured Human Podocytes.” Journal of Cellular and Molecular Medicine 19, no. 12: 2771–2779. 10.1111/jcmm.12660. 26404773 PMC4687697

[jcp70116-bib-0003] Artunc, F. , E. Schleicher , C. Weigert , A. Fritsche , N. Stefan , and H. U. Häring . 2016. “The Impact of Insulin Resistance on the Kidney and Vasculature.” Nature Reviews Nephrology 12, no. 12: 721–737. 10.1038/nrneph.2016.145. 27748389

[jcp70116-bib-0004] Audzeyenka, I. , P. Rachubik , D. Rogacka , et al. 2020. “Cathepsin C Is a Novel Mediator of Podocyte and Renal Injury Induced by Hyperglycemia.” Biochimica et Biophysica Acta (BBA) ‐ Molecular Cell Research 1867, no. 8: 118723. 10.1016/j.bbamcr.2020.118723. 32302668

[jcp70116-bib-0005] Benarafa, C. 2015. “Regulation of Neutrophil Serine Proteases by Intracellular Serpins.” In The Serpin Family: Proteins With Multiple Functions in Health and Disease, 59–76. 10.1007/978-3-319-22711-5_5.

[jcp70116-bib-0006] Benarafa, C. , G. P. Priebe , and E. Remold‐O'donnell . 2007. “The Neutrophil Serine Protease Inhibitor serpinb1 Preserves Lung Defense Functions in Pseudomonas Aeruginosa Infection.” Journal of Experimental Medicine 204, no. 8: 1901–1909. 10.1084/jem.20070494. 17664292 PMC2118684

[jcp70116-bib-0007] Benarafa, C. , and H. U. Simon . 2017. “Role of Granule Proteases in the Life and Death of Neutrophils.” Biochemical and Biophysical Research Communications 482, no. 3: 473–481. 10.1016/j.bbrc.2016.11.086. 28212734

[jcp70116-bib-0008] Bollée, G. , M. Flamant , S. Schordan , et al. 2011. “Epidermal Growth Factor Receptor Promotes Glomerular Injury and Renal Failure in Rapidly Progressive Crescentic Glomerulonephritis.” Nature Medicine 17, no. 10: 1242–1250. 10.1038/nm.2491. PMC3198052 21946538

[jcp70116-bib-0009] Bronze‐Da‐Rocha, E. , and A. Santos‐Silva . 2018. “Neutrophil Elastase Inhibitors and Chronic Kidney Disease.” International Journal of Biological Sciences 14, no. 10: 1343–1360. 10.7150/ijbs.26111. 30123081 PMC6097478

[jcp70116-bib-0010] Burgener, S. S. , N. G. F. Leborgne , S. J. Snipas , G. S. Salvesen , P. I. Bird , and C. Benarafa . 2019. “Cathepsin G Inhibition by Serpinb1 and Serpinb6 Prevents Programmed Necrosis in Neutrophils and Monocytes and Reduces Gsdmd‐Driven Inflammation.” Cell Reports 27, no. 12: 3646–3656.e5. 10.1016/j.celrep.2019.05.065. 31216481 PMC7350907

[jcp70116-bib-0011] Caster, D. J. , E. A. Korte , M. Tan , et al. 2018. “Neutrophil Exocytosis Induces Podocyte Cytoskeletal Reorganization and Proteinuria in Experimental Glomerulonephritis.” American Journal of Physiology ‐ Renal Physiology 315, no. 3: F595–F606. 10.1152/ajprenal.00039.2018. 29790391 PMC6172569

[jcp70116-bib-0012] Cavallari, J. F. , M. D. Fullerton , B. M. Duggan , et al. 2017. “Muramyl Dipeptide‐Based Postbiotics Mitigate Obesity‐Induced Insulin Resistance via IRF4.” Cell Metabolism 25, no. 5: 1063–1074.e3. 10.1016/j.cmet.2017.03.021. 28434881

[jcp70116-bib-0013] Chalmers, J. D. , R. Kettritz , and B. Korkmaz . 2023. “Dipeptidyl Peptidase 1 Inhibition as a Potential Therapeutic Approach in Neutrophil‐Mediated Inflammatory Disease.” Frontiers in Immunology 14: 1239151. 10.3389/fimmu.2023.1239151. 38162644 PMC10755895

[jcp70116-bib-0014] Chua, F. 2006. “Neutrophil Elastase: Mediator of Extracellular Matrix Destruction and Accumulation.” Proceedings of the American Thoracic Society 3, no. 5: 424–427. 10.1513/pats.200603-078AW. 16799086

[jcp70116-bib-0015] Coward, R. , and A. Fornoni . 2015. “Insulin Signaling: Implications for Podocyte Biology in Diabetic Kidney Disease.” Current Opinion in Nephrology and Hypertension 24, no. 1: 104–110. 10.1097/MNH.0000000000000078. 25415617 PMC4386894

[jcp70116-bib-0016] Davies, M. , A. J. Barrett , J. Travis , E. Sanders , and G. A. Coles . 1978. “The Degradation of Human Glomerular Basement Membrane With Purified Lysosomal Proteinases: Evidence for the Pathogenic Role of the Polymorphonuclear Leucocyte in Glomerulonephritis.” Clinical Science and Molecular Medicine 54, no. 3: 233–240. 10.1042/cs0540233. 630800

[jcp70116-bib-0017] Denou, E. , K. Lolmède , L. Garidou , et al. 2015. “Defective NOD2 Peptidoglycan Sensing Promotes Diet‐Induced Inflammation, Dysbiosis, and Insulin Resistance.” EMBO Molecular Medicine 7, no. 3: 259–274. 10.15252/emmm.201404169. 25666722 PMC4364944

[jcp70116-bib-0018] D'Souza, R. F. , S. W. C. Masson , J. S. T. Woodhead , et al. 2021. “α1‐Antitrypsin A Treatment Attenuates Neutrophil Elastase Accumulation and Enhances Insulin Sensitivity in Adipose Tissue of Mice Fed a High‐Fat Diet.” American Journal of Physiology ‐ Endocrinology and Metabolism 321, no. 4: E560–E570. 10.1152/ajpendo.00181.2021. 34486403

[jcp70116-bib-0019] Du, P. , B. Fan , H. Han , et al. 2013. “NOD2 Promotes Renal Injury by Exacerbating Inflammation and Podocyte Insulin Resistance in Diabetic Nephropathy.” Kidney International 84, no. 2: 265–276. 10.1038/ki.2013.113. 23594678

[jcp70116-bib-0020] Faul, C. 2014. “The Podocyte Cytoskeleton: Key to a Functioning Glomerulus in Health and Disease.” In Podocytopathy, edited by Z.‐H. Liu , and J. C. He , Vol. 183, 22–53. 10.1159/000359923.

[jcp70116-bib-0021] Feith, G. W. , K. J. M. Assmann , M. J. J. T. Bogman , A. P. M. Van Gompel , J. Schalkwijk , and R. A. P. Koene . 1996. “Different Mediator Systems in Biphasic Heterologous Phase of Anti‐GBM Nephritis in Mice.” Nephrology Dialysis Transplantation 11, no. 4: 599–607. 10.1093/oxfordjournals.ndt.a027347. 8671846

[jcp70116-bib-0022] Fukuta, M. , K. Suzuki , S. Kojima , et al. 2021. “Suppressor of Cytokine Signalling 3 (SOCS3) Expressed in Podocytes Attenuates Glomerulonephritis and Suppresses Autoantibody Production in an Imiquimod‐Induced Lupus Model.” Lupus Science & Medicine 8, no. 1: e000426. 10.1136/lupus-2020-000426. 34016718 PMC8141454

[jcp70116-bib-0023] Funk, J. , V. Ott , A. Herrmann , et al. 2016. “Semiautomated Quantitative Image Analysis of Glomerular Immunohistochemistry Markers Desmin, Vimentin, Podocin, Synaptopodin and WT‐1 in Acute and Chronic Rat Kidney Disease Models.” Histochemistry and Cell Biology 145, no. 3: 315–326. 10.1007/s00418-015-1391-6. 26671788

[jcp70116-bib-0024] Garratt, L. W. , E. N. Sutanto , K. M. Ling , et al. 2015. “Matrix Metalloproteinase Activation by Free Neutrophil Elastase Contributes to Bronchiectasis Progression in Early Cystic Fibrosis.” European Respiratory Journal 46, no. 2: 384–394. 10.1183/09031936.00212114. 25929954

[jcp70116-bib-0025] Garsen, M. , A. L. W. M. M. Rops , H. Dijkman , et al. 2016. “Cathepsin L Is Crucial for the Development of Early Experimental Diabetic Nephropathy.” Kidney International 90, no. 5: 1012–1022. 10.1016/j.kint.2016.06.035. 27575559

[jcp70116-bib-0026] Giambelluca, S. , M. Ochs , and E. Lopez‐Rodriguez . 2022. “Resting Time After Phorbol 12‐myristate 13‐acetate in THP‐1 Derived Macrophages Provides a Non‐Biased Model for the Study of NLRP3 Inflammasome.” Frontiers in Immunology 13: 958098. 10.3389/fimmu.2022.958098. 36618426 PMC9817155

[jcp70116-bib-0027] Gluhovschi, G. , C. Gluhovschi , F. Bob , et al. 2010. “Immune Compartments of the Nephron in Relation to the Immune System.” Romanian Journal of Internal Medicine 48, no. 1: 17–31. 21180237

[jcp70116-bib-0028] Goldwich, A. , M. Burkard , M. Ölke , et al. 2013. “Podocytes Are Nonhematopoietic Professional Antigen‐Presenting Cells.” Journal of the American Society of Nephrology 24, no. 6: 906–916. 10.1681/ASN.2012020133. 23539760 PMC3665387

[jcp70116-bib-0029] Gough, N. R. 2012. “Neutrophils Suppress Insulin Signaling.” Science Signaling 5, no. 243: ec250. 10.1126/scisignal.2003633.

[jcp70116-bib-0030] Guan, Y. , D. Nakano , Y. Zhang , et al. 2017. “A Protease‐Activated receptor‐1 Antagonist Protects Against Podocyte Injury in a Mouse Model of Nephropathy.” Journal of Pharmacological Sciences 135, no. 2: 81–88. 10.1016/j.jphs.2017.09.002. 29110957

[jcp70116-bib-0031] Hale, L. J. , and R. J. M. Coward . 2013. “The Insulin Receptor and the Kidney.” Current Opinion in Nephrology and Hypertension 22, no. 1: 100–106. 10.1097/MNH.0b013e32835abb52. 23104093

[jcp70116-bib-0032] Han, H. , Y. Wang , X. Li , et al. 2013. “Novel Role of NOD2 in Mediating Ca2+ Signaling: Evidence From NOD2‐regulated Podocyte TRPC6 Channels in Hyperhomocysteinemia.” Hypertension 62, no. 3: 506–511. 10.1161/HYPERTENSIONAHA.113.01638. 23856489

[jcp70116-bib-0033] Höhne, M. , C. K. Frese , F. Grahammer , et al. 2018. “Single‐Nephron Proteomes Connect Morphology and Function in Proteinuric Kidney Disease.” Kidney International 93, no. 6: 1308–1319. 10.1016/j.kint.2017.12.012. 29530281

[jcp70116-bib-0034] Jablaoui, A. , A. Kriaa , H. Mkaouar , et al. 2020. “Fecal Serine Protease Profiling in Inflammatory Bowel Diseases.” Frontiers in Cellular and Infection Microbiology 10: 21. 10.3389/fcimb.2020.00021. 32117798 PMC7011180

[jcp70116-bib-0035] Jauregui, A. , D. H. Mintz , P. Mundel , and A. Fornoni . 2009. “Role of Altered Insulin Signaling Pathways in the Pathogenesis of Podocyte Malfunction and Microalbuminuria.” Current Opinion in Nephrology and Hypertension 18, no. 6: 539–545. 10.1097/MNH.0b013e32832f7002. 19724224 PMC2907246

[jcp70116-bib-0036] Jégot, G. , C. Derache , S. Castella , et al. 2011. “A Substrate‐Based Approach to Convert SerpinB1 Into a Specific Inhibitor of Proteinase 3, the Wegener's Granulomatosis Autoantigen.” FASEB Journal 25, no. 9: 3019–3031. 10.1096/fj.10-176552. 21670065

[jcp70116-bib-0037] Jeong, I. , J. K. Yun , J. O. Jin , et al. 2024. “E3 Ligase SOCS3 Regulates NOD2 Expression by Ubiquitin Proteasome System in Lung Cancer Progression.” Cellular Oncology 47, no. 3: 819–832. 10.1007/s13402-023-00896-5. 37910276 PMC12974036

[jcp70116-bib-0038] Johnson, R. J. , W. G. Couser , E. Y. Chi , S. Adler , and S. J. Klebanoff . 1987. “New Mechanism for Glomerular Injury. Myeloperoxidase‐Hydrogen Peroxide‐Halide System.” Journal of Clinical Investigation 79, no. 5: 1379–1387. 10.1172/JCI112965. 3033023 PMC424393

[jcp70116-bib-0039] Kaufman, D. P. , H. Basit , and S. J. Knohl . 2023. “Physiology, Glomerular Filtration Rate.” In StatPearls. 29763208

[jcp70116-bib-0040] Keestra‐Gounder, A. M. , and R. M. Tsolis . 2017. “NOD1 and NOD2: Beyond Peptidoglycan Sensing.” In Trends in Immunology 38, no. 10: 758–767. 10.1016/j.it.2017.07.004. PMC5624830 28823510

[jcp70116-bib-0041] Kulesza, T. , M. Typiak , P. Rachubik , et al. 2022. “Hyperglycemic Environment Disrupts Phosphate Transporter Function and Promotes Calcification Processes in Podocytes and Isolated Glomeruli.” Journal of Cellular Physiology 237, no. 5: 2478–2491. 10.1002/jcp.30700. 35150131

[jcp70116-bib-0042] Kuravi, S. J. , H. M. McGettrick , S. C. Satchell , et al. 2014. “Podocytes Regulate Neutrophil Recruitment by Glomerular Endothelial Cells via IL‐6–Mediated Crosstalk.” Journal of Immunology 193, no. 1: 234–243. 10.4049/jimmunol.1300229. PMC4067868 24872191

[jcp70116-bib-0043] Kuźniar, J. , T. J. Kuźniar , Z. Marchewka , et al. 2007. “Elastase Deposits in the Kidney and Urinary Elastase Excretion in Patients With Glomerulonephritis ‐ Evidence for Neutrophil Involvement in Renal Injury.” Scandinavian Journal of Urology and Nephrology 41, no. 6: 527–534. 10.1080/00365590701430893. 17853021

[jcp70116-bib-0044] Kyohara, M. , D. Miyashita , R. Inoue , et al. 2022. “Association Between Circulating SerpinB1 Levels and Insulin Sensitivity in Japanese With Type 2 Diabetes: A Single‐Center, Crosssectional, Observational Study.” PLoS ONE 17, no. 11: e0276915. 10.1371/journal.pone.0276915. 36331940 PMC9635728

[jcp70116-bib-0045] Lee, E. B. , A. Kim , K. Kang , H. Kim , and J.‐S. Lim . 2010. “NDRG2‐mediated Modulation of SOCS3 and STAT3 Activity Inhibits IL‐10 Production.” Immune Network 10, no. 6: 219. 10.4110/in.2010.10.6.219. 21286383 PMC3026942

[jcp70116-bib-0046] Lee, K. H. , A. Biswas , Y. J. Liu , and K. S. Kobayashi . 2012. “Proteasomal Degradation of Nod2 Protein Mediates Tolerance to Bacterial Cell Wall Components.” Journal of Biological Chemistry 287, no. 47: 39800–39811. 10.1074/jbc.M112.410027. 23019338 PMC3501029

[jcp70116-bib-0047] Lessieur, E. M. , H. Liu , A. Saadane , et al. 2021. “Neutrophil‐Derived Proteases Contribute to the Pathogenesis of Early Diabetic Retinopathy.” Investigative Opthalmology & Visual Science 62, no. 13: 7. 10.1167/iovs.62.13.7. PMC8525836 34643662

[jcp70116-bib-0048] Li, J. Z. , R. Sharma , K. N. Dileepan , and V. J. Savin . 1994. “Polymorphonuclear Leukocytes Increase Glomerular Albumin Permeability via Hypohalous Acid.” Kidney International 46, no. 4: 1025–1030. 10.1038/ki.1994.363. 7861697

[jcp70116-bib-0049] Li, K. , L. Dong , S. Gao , et al. 2024. “Safety, Tolerability, Pharmacokinetics and Neutrophil Elastase Inhibitory Effects of Sivelestat: A Randomized, Double‐Blind, Placebo‐Controlled Single‐ and Multiple‐Dose Escalation Study in Chinese Healthy Subjects.” European Journal of Pharmaceutical Sciences 195: 106723. 10.1016/j.ejps.2024.106723. 38336251

[jcp70116-bib-0050] Li, S. , Y. Liu , Y. He , et al. 2020. “Podocytes Present Antigen to Activate Specific T Cell Immune Responses in Inflammatory Renal Disease.” Journal of Pathology 252, no. 2: 165–177. 10.1002/path.5508. 32686090

[jcp70116-bib-0051] Liu, Y. , H. Hitomi , S. Diah , et al. 2013. “Roles of Na+/H+ Exchanger Type 1 and Intracellular Ph in Angiotensin Ii‐Induced Reactive Oxygen Species Generation and Podocyte Apoptosis.” Journal of Pharmacological Sciences 122, no. 3: 176–183. 10.1254/jphs.12291FP. 23800993 PMC3792360

[jcp70116-bib-0052] Majewski, P. , M. Majchrzak‐Gorecka , B. Grygier , J. Skrzeczynska‐Moncznik , O. Osiecka , and J. Cichy . 2016. “Inhibitors of Serine Proteases in Regulating the Production and Function of Neutrophil Extracellular Traps.” Frontiers in Immunology 7, no. JUN: 261. 10.3389/fimmu.2016.00261. 27446090 PMC4928128

[jcp70116-bib-0053] Maurya, C. K. , D. Arha , A. K. Rai , et al. 2015. “NOD2 Activation Induces Oxidative Stress Contributing to Mitochondrial Dysfunction and Insulin Resistance in Skeletal Muscle Cells.” Free Radical Biology and Medicine 89: 158–169. 10.1016/j.freeradbiomed.2015.07.154. 26404168

[jcp70116-bib-0054] Mirea, A. M. , E. J. M. Toonen , I. Van Den Munckhof , et al. 2019. “Increased Proteinase 3 and Neutrophil Elastase Plasma Concentrations Are Associated With Non‐Alcoholic Fatty Liver Disease (NAFLD) and Type 2 Diabetes.” Molecular Medicine 25, no. 1: 16. 10.1186/s10020-019-0084-3. 31046673 PMC6498541

[jcp70116-bib-0055] Mohanan, V. , and C. L. Grimes . 2014. “The Molecular Chaperone HSP70 Binds to and Stabilizes NOD2, an Important Protein Involved in Crohn Disease.” Journal of Biological Chemistry 289, no. 27: 18987–18998. 10.1074/jbc.M114.557686. 24790089 PMC4081938

[jcp70116-bib-0056] Mukherjee, S. , J. H. Graber , and C. L. Moore . 2023. “Macrophage Differentiation Is Marked by Increased Abundance of the mRNA 3′ End Processing Machinery, Altered Poly(A) Site Usage, and Sensitivity to the Level of CstF64.” Frontiers in Immunology 14: 1091403. 10.3389/fimmu.2023.1091403. 36761770 PMC9905730

[jcp70116-bib-0057] Münzel, T. , S. Kurz , S. Rajagopalan , et al. 1996. “Hydralazine Prevents Nitroglycerin Tolerance by Inhibiting Activation of a Membrane‐Bound NADH Oxidase: A New Action for an Old Drug.” Journal of Clinical Investigation 98, no. 6: 1465–1470. 10.1172/JCI118935. 8823313 PMC507574

[jcp70116-bib-0058] Mutua, V. , and L. J. Gershwin . 2021. “A Review of Neutrophil Extracellular Traps (NETs) in Disease: Potential Anti‐NETs Therapeutics.” Clinical Reviews in Allergy and Immunology 61, no. 2: 194–211. 10.1007/s12016-020-08804-7. 32740860 PMC7395212

[jcp70116-bib-0059] Oda, T. , O. Hotta , Y. Taguma , et al. 1997. “Involvement of Neutrophil Elastase in Crescentic Glomerulonephritis.” Human Pathology 28, no. 6: 720–728. 10.1016/S0046-8177(97)90182-9. 9191007

[jcp70116-bib-0060] Othman, A. , M. Sekheri , and J. G. Filep . 2022. “Roles of Neutrophil Granule Proteins in Orchestrating Inflammation and Immunity.” FEBS Journal 289, no. 14: 3932–3953. 10.1111/febs.15803. 33683814 PMC9546106

[jcp70116-bib-0061] El Ouaamari, A. , E. Dirice , N. Gedeon , et al. 2016. “SerpinB1 Promotes Pancreatic β Cell Proliferation.” Cell Metabolism 23, no. 1: 194–205. 10.1016/j.cmet.2015.12.001. 26701651 PMC4715773

[jcp70116-bib-0062] Park, K. A. , H. S. Byun , M. Won , et al. 2007. “Sustained Activation of Protein Kinase C Downregulates Nuclear factor‐κ B Signaling by Dissociation of IKK‐γ and Hsp90 Complex in Human Colonic Epithelial Cells.” Carcinogenesis 28, no. 1: 71–80. 10.1093/carcin/bgl094. 16774932

[jcp70116-bib-0063] Piwkowska, A. , D. Rogacka , M. Jankowski , and S. Angielski . 2013. “Metformin Reduces NAD(P)H Oxidase Activity in Mouse Cultured Podocytes Through Purinergic Dependent Mechanism by Increasing Extracellular ATP Concentration.” Acta Biochimica Polonica 60, no. 4: 607–612. 10.18388/abp.2013_2028. 24432311

[jcp70116-bib-0064] Piwkowska, A. , D. Rogacka , M. Jankowski , M. H. Dominiczak , J. K. Stępiński , and S. Angielski . 2010. “Metformin Induces Suppression of NAD(P)H Oxidase Activity in Podocytes.” Biochemical and Biophysical Research Communications 393, no. 2: 268–273. 10.1016/j.bbrc.2010.01.119. 20123087

[jcp70116-bib-0065] Piwkowska, A. , D. Rogacka , M. Jankowski , K. Kocbuch , and S. Angielski . 2012. “Hydrogen Peroxide Induces Dimerization of Protein Kinase G Type Iα Subunits and Increases Albumin Permeability in Cultured Rat Podocytes.” Journal of Cellular Physiology 227, no. 3: 1004–1016. 10.1002/jcp.22810. 21520075

[jcp70116-bib-0066] Pollak, M. R. , S. E. Quaggin , M. P. Hoenig , and L. D. Dworkin . 2014. “The Glomerulus: The Sphere of Influence.” Clinical Journal of the American Society of Nephrology 9, no. 8: 1461–1469. 10.2215/CJN.09400913. 24875196 PMC4123398

[jcp70116-bib-0067] Popow‐Stellmaszyk, J. , M. Wysocka , A. Lesner , B. Korkmaz , and K. Rolka . 2013. “A New Proteinase 3 Substrate With Improved Selectivity over Human Neutrophil Elastase.” Analytical Biochemistry 442, no. 1: 75–82. 10.1016/j.ab.2013.07.028. 23911525

[jcp70116-bib-0068] Potempa, J. , E. Korzus , and J. Travis . 1994. “The Serpin Superfamily of Proteinase Inhibitors: Structure, Function, and Regulation.” Journal of Biological Chemistry 269, no. 23: 15957–15960. 10.1016/s0021-9258(17)33954-6. 8206889

[jcp70116-bib-0069] Rinschen, M. M. , P. F. Huesgen , and R. E. Koch . 2018. “The Podocyte Protease Web: Uncovering the Gatekeepers of Glomerular Disease.” American Journal of Physiology‐Renal Physiology 315, no. 6: F1812–F1816. 10.1152/ajprenal.00380.2018. 30230368

[jcp70116-bib-0070] Rogacka, D. 2021. “Insulin Resistance in Glomerular Podocytes: Potential Mechanisms of Induction.” Archives of Biochemistry and Biophysics 710, no. March: 109005. 10.1016/j.abb.2021.109005. 34371008

[jcp70116-bib-0071] Rogacka, D. , A. Piwkowska , I. Audzeyenka , S. Angielski , and M. Jankowski . 2014. “Involvement of the AMPK‐PTEN Pathway in Insulin Resistance Induced by High Glucose in Cultured Rat Podocytes.” International Journal of Biochemistry & Cell Biology 51: 120–130. 10.1016/j.biocel.2014.04.008. 24747132

[jcp70116-bib-0072] Saleem, M. A. , M. J. O'Hare , J. Reiser , et al. 2002. “A Conditionally Immortalized Human Podocyte Cell Line Demonstrating Nephrin and Podocin Expression.” Journal of the American Society of Nephrology 13, no. 3: 630–638. 11856766 10.1681/ASN.V133630

[jcp70116-bib-0073] Schrijver, G. , J. Schalkwijk , J. C. Robben , K. J. Assmann , and R. A. Koene . 1989. “Antiglomerular Basement Membrane Nephritis in Beige Mice. Deficiency of Leukocytic Neutral Proteinases Prevents the Induction of Albuminuria in the Heterologous Phase.” Journal of Experimental Medicine 169, no. 4: 1435–1448. 10.1084/jem.169.4.1435. 2538553 PMC2189245

[jcp70116-bib-0074] Sever, S. , M. M. Altintas , S. R. Nankoe , et al. 2007. “Proteolytic Processing of Dynamin by Cytoplasmic Cathepsin L Is a Mechanism for Proteinuric Kidney Disease.” Journal of Clinical Investigation 117, no. 8: 2095–2104. 10.1172/JCI32022. 17671649 PMC1934589

[jcp70116-bib-0075] Silverman, G. A. , P. I. Bird , R. W. Carrell , et al. 2001. “The Serpins Are an Expanding Superfamily of Structurally Similar but Functionally Diverse Proteins. Evolution, Mechanism of Inhibition, Novel Functions, and a Revised Nomenclature.” Journal of Biological Chemistry 276, no. 36: 33293–33296. 10.1074/jbc.R100016200. 11435447

[jcp70116-bib-0076] Stevens, C. , P. Henderson , E. R. Nimmo , et al. 2013. “The Intermediate Filament Protein, Vimentin, Is a Regulator of NOD2 Activity.” Gut 62, no. 5: 695–707. 10.1136/gutjnl-2011-301775. 22684479 PMC4225453

[jcp70116-bib-0077] Suzuki, S. , F. Gejyo , T. Kuroda , et al. 1998. “Effects of a Novel Elastase Inhibitor, ONO‐5046, on Nephrotoxic Serum Nephritis in Rats.” Kidney International 53, no. 5: 1201–1208. 10.1046/j.1523-1755.1998.00872.x. 9573534

[jcp70116-bib-0078] Takebayashi, K. , K. Hara , T. Terasawa , et al. 2016. “Circulating SerpinB1 Levels and Clinical Features in Patients With Type 2 Diabetes.” BMJ Open Diabetes Research & Care 4, no. 1: e000274. 10.1136/bmjdrc-2016-000274. PMC5128937 27933185

[jcp70116-bib-0079] Talukdar, S. , D. Y. Oh , G. Bandyopadhyay , et al. 2012. “Neutrophils Mediate Insulin Resistance in Mice Fed a High‐Fat Diet Through Secreted Elastase.” Nature Medicine 18, no. 9: 1407–1412. 10.1038/nm.2885. PMC3491143 22863787

[jcp70116-bib-0080] Tamrakar, A. K. , J. D. Schertzer , T. T. Chiu , et al. 2010. “NOD2 Activation Induces Muscle Cell‐Autonomous Innate Immune Responses and Insulin Resistance.” Endocrinology 151, no. 12: 5624–5637. 10.1210/en.2010-0437. 20926588

[jcp70116-bib-0081] Terstegen, L. , B. G. Maassen , S. Radtke , et al. 2000. “Differential Inhibition of IL‐6‐type Cytokine‐Induced STAT Activation by PMA.” FEBS Letters 478, no. 1–2: 100–104. 10.1016/S0014-5793(00)01826-3. 10922477

[jcp70116-bib-0082] Wang, L. Y. , X. J. Sun , M. Chen , and M. H. Zhao . 2019. “The Expression of NOD2, NLRP3 and NLRC5 and Renal Injury in Anti‐Neutrophil Cytoplasmic Antibody‐Associated Vasculitis.” Journal of Translational Medicine 17, no. 1: 197. 10.1186/s12967-019-1949-5. 31186034 PMC6560890

[jcp70116-bib-0083] Wang, Y. , D. Lu , S. Lv , X. Liu , and G. Liu . 2024. “Mesenchymal Stem Cell‐Derived Exosomes Ameliorate Diabetic Kidney Disease Through NOD2 Signaling Pathway.” Renal Failure 46, no. 2: 2381597. 10.1080/0886022X.2024.2381597. 39039856 PMC11268218

[jcp70116-bib-0084] Wang, Y. , Y. Xiao , L. Zhong , et al. 2014. “Increased Neutrophil Elastase and Proteinase 3 and Augmented NETosis Are Closely Associated With β‐cell Autoimmunity in Patients With Type 1 Diabetes.” Diabetes 63, no. 12: 4239–4248. 10.2337/db14-0480. 25092677

[jcp70116-bib-0085] Williams, L. , A. Alshehri , B. Robichaud , A. Cudmore , and J. Gagnon . 2020. “The Role of the Bacterial Muramyl Dipeptide in the Regulation of GLP‐1 and Glycemia.” International Journal of Molecular Sciences 21, no. 15: 5252. 10.3390/ijms21155252. 32722085 PMC7432949

[jcp70116-bib-0086] Yaddanapudi, S. , M. M. Altintas , A. D. Kistler , et al. 2011. “CD2AP in Mouse and Human Podocytes Controls a Proteolytic Program That Regulates Cytoskeletal Structure and Cellular Survival.” Journal of Clinical Investigation 121, no. 10: 3965–3980. 10.1172/JCI58552. 21911934 PMC3195478

[jcp70116-bib-0087] Yamamoto‐Nonaka, K. , M. Koike , K. Asanuma , et al. 2016. “Cathepsin D in Podocytes Is Important in the Pathogenesis of Proteinuria and CKD.” Journal of the American Society of Nephrology 27, no. 9: 2685–2700. 10.1681/ASN.2015040366. 26823550 PMC5004641

[jcp70116-bib-0088] Zhirnov, O. P. , H. D. Klenk , and P. F. Wright . 2011. “Aprotinin and Similar Protease Inhibitors as Drugs Against Influenza.” Antiviral Research 92, no. 1: 27–36. 10.1016/j.antiviral.2011.07.014. 21802447

